# MGMA-PPIS: Predicting the protein–protein interaction site with multiview graph embedding and multiscale attention fusion

**DOI:** 10.1093/gigascience/giaf114

**Published:** 2025-10-01

**Authors:** Yong Han, Shao-Wu Zhang, Qing-Qing Zhang, Ming-Hui Shi

**Affiliations:** MOE Key Laboratory of Information Fusion Technology, School of Automation, Northwestern Polytechnical University, Xi’an 710072, China; Department of Police Administration, Henan Judicial Police Vocational College, Zhengzhou 450046, China; MOE Key Laboratory of Information Fusion Technology, School of Automation, Northwestern Polytechnical University, Xi’an 710072, China; MOE Key Laboratory of Information Fusion Technology, School of Automation, Northwestern Polytechnical University, Xi’an 710072, China; MOE Key Laboratory of Information Fusion Technology, School of Automation, Northwestern Polytechnical University, Xi’an 710072, China

**Keywords:** protein–protein interaction sites, graph neural network, multiscale attention network

## Abstract

**Background:**

Protein–protein interactions (PPIs) play a crucial role in numerous biological processes. Accurate identification of protein–protein interaction sites is critical for a comprehensive understanding of protein functions and pathological mechanisms. However, conventional experimental approaches for detecting PPIs are often time-consuming and labor-intensive, thereby motivating the development of efficient computational methods to identify PPI sites.

**Results:**

In this work, we propose a novel graph neural network–based method (called MGMA-PPIS) to predict PPI sites by adopting multiview graph embedding and multiscale attention fusion. MGMA-PPIS integrates global node features extracted by an equivariant graph neural network and multiscale local node features extracted by an edge graph attention network across different neighborhood scales, thereby constructing a multiview graph feature representation. Then, a multiscale attention network is employed to perform deep feature fusion across multiple scales for achieving high-precision prediction of PPI sites.

**Conclusions:**

Experimental results on benchmark datasets show that our MGMA-PPIS outperforms other state-of-the-art methods, and it can effectively predict PPI sites.

## Introduction

Proteins perform a wide variety of cellular functions within cells and play key roles in diverse biological processes such as signal transduction, transport, and metabolism [1]. However, proteins rarely function alone; in over 80% of cases, they act as part of complexes [2]. Therefore, studying protein–protein interactions (PPIs) can help build protein interaction networks [3], predict protein functions [4], provide insights into disease mechanisms [5], and pave the way for drug development and design [6]. PPI sites refer to the interface residues of proteins involved in these interactions. Identifying these sites is crucial for unraveling cellular processes and advancing novel drug discovery [7]. However, experimental identification of PPI sites through wet-lab methods, such as coimmunoprecipitation [8] and 2-hybrid screening [9], is increasingly impractical due to high time and cost demands. Consequently, it is particularly necessary to develop efficient computational methods as powerful guides and complements to genetic and biochemical experiments.

To date, a variety of computational methods have been developed to predict PPI sites. Early PPI site prediction approaches primarily relied on machine learning, such as naive Bayes classifiers [10], LightGBM [11], random forests [12, 13], and XGBoost [14, 15]. These methods employ the feature engineering to select appropriate features, such as raw protein sequences, position-specific scoring matrices (PSSMs), and definitions of secondary protein structures (DSSP), to represent proteins, and then use machine learning algorithms to predict PPI sites. In recent years, researchers have increasingly turned their attention to deep learning algorithms, which have further improved prediction accuracy of PPI sites [16, 17]. Existing deep learning methods for predicting PPI sites can be broadly categorized into sequence-based methods (e.g., convolutional neural networks and recurrent neural networks) and structure-based methods (e.g., graph neural networks). Convolutional neural network (CNN)–based methods, such as DeepPPISP [17] and ProB-site [18], capture the local contextual features of protein sequences through convolution operations to predict PPI sites. DeepPPISP [17] first extracts the local contextual features from the neighboring amino acids of a target residue by using a sliding window approach and then extracts the global features from protein sequences using TextCNN. Then, the local contextual features and the global features are integrated together and fed them into a fully connected (FC) layer for predicting PPI sites. ProB-site [18] utilizes sub-CNN architecture to extract 3 separate higher-order features from sequential information of proteins and feeds them into the FC layer to achieve accurate prediction of PPI sites. Although these CNN-based methods perform well, they often overlook the hidden long-range dependencies within protein sequences. In contrast, recurrent neural network (RNN)–based methods, such as DELPHI [19] and DLPred [20], are capable of handling long-range dependencies and global information from protein sequences, enabling more robust representation of long-distance correlations in protein sequences. DELPHI [19] uses the high-scoring segment pairs (HSPs), position information, and 3-mer amino acid embedding as 3 novel features and then builds an ensemble framework with the CNN and RNN for predicting PPI sites. DLPred [20] employs a deep learning architecture based on simplified long short-term memory (SLSTM) networks to enhance the prediction performance of PPI sites. With the emergence of graph neural networks (GNNs), structure-based PPI site prediction methods that utilize protein tertiary structural features have advanced, enabling the accurate extraction of protein features from protein structures. In particular, the growing availability of protein tertiary structure data and the advent of high-accuracy structure prediction tools like AlphaFold2 [21] have significantly advanced the application of GNNs in PPI site prediction, yielding some PPI site prediction methods, such as AGAT-PPIS [16], GraphPPIS [22], AGF-PPIS [23], and GHGPR-PPIS [24]. AGAT-PPIS [16] integrates edge features to calculate attention scores for refining node embeddings, thereby facilitating the prediction of PPI sites. GraphPPIS [22] adopts a deep graph convolutional neural network (GCN) framework to predict PPI sites by incorporating initial residual connections and identity mapping techniques. AGF-PPIS [23] leverages the multihead self-attention mechanisms, graph convolutional networks, and feedforward neural networks to extract protein features, which are subsequently inputted into a multilayer perceptron (MLP) for achieving PPI site prediction. GHGPR-PPIS [24] first constructs a graph network by using a heat kernel-based graph convolutional network, then combines the generalized PageRank approach with an edge self-attention feature-processing trick to extract features that are inputted into an MLP for PPI sites prediction. Although the above GNN-based methods have achieved good performance, they often rely on a single encoder, which hinders their ability to comprehensively extract information from complex proteins. Meanwhile, with the rise of large language models such as the classic Transformer [25], attention mechanisms have garnered increasing attention, which are used to enhance the performance of GCNs [26]. However, they employ a traditional self-attention mechanism, which often encounters problems such as excessive concentration or dispersion of attention [27], resulting in inadequate information representation, thereby hindering the model’s ability to accurately interpret the original input.

In this work, we propose a novel computational method (named MGMA-PPIS) to predict PPI sites by introducing multiview graph embeddings and multiscale attention-based feature fusion mechanism. MGMA-PPIS aims to enhance existing GNN-based methods for high-precision prediction of PPI sites. Specifically, MGMA-PPIS first constructs an adjacency matrix for the protein graph by calculating the Euclidean distances between amino acids and integrates both sequence and structure information to generate representations for nodes and edges. Subsequently, the multirange local and global embeddings of protein features are processed by the edge graph attention network (EGAT) and E(n) equivariant graph neural network (EGNN), respectively, and finally are integrated through a multiscale attention mechanism to achieve feature embedding fusion, enabling more effective capture of key information. Moreover, MGMA-PPIS utilizes the focal loss function [23, 28] to optimize the model and mitigate the impact of class imbalance. To the best of our knowledge, this is the first attempt to apply multiview graph embedding fusion to PPI site prediction. Comprehensive evaluations on multiple benchmark datasets and independent test sets demonstrate that MGMA-PPIS significantly outperforms other existing methods.

The key innovation of our MGMA-PPIS lies in the synergistic combination of an EGAT and an EGNN for complementary local and global protein feature extraction, coupled with a parallel multiscale attention fusion strategy at the amino acid level. Specifically, EGAT incorporates edge features to capture fine-grained local patterns across multiple neighborhood scales, while EGNN preserves E(n) equivariance (translation, rotation, reflection, and permutation) in extracting robust global features from the overall spatial structure. Unlike conventional self-attention, which models dependencies at a single scale, the proposed multiscale attention mechanism enables simultaneous multiscale context modeling, thereby enhancing predictive accuracy and fully exploiting the complementarity of local and global information.

## Materials and Methods

### Datasets

In this work, we utilize the same benchmark dataset as previous work on AGAT-PPIS [16] for parameter tuning and model performance testing. The AGAT-PPIS dataset is derived from the GraphPPIS dataset, which includes the following subsets: 1 training set of Train_335–1 and 3 test sets of Test_315–28, Test_60–0, and Ubtest_31–6. The statistical information of the AGAT-PPIS datasets is shown in Supplementary Table S1.

### Protein representation

In the MGMA-PPIS framework, we use an undirected graph **G** = (**V, A, E**) to represent each protein. Here, ${{\bf V}}{\mathrm{ = }}\{ {{{v}_i}} \}$ is the amino acid residue node set, ${{v}_i} \in {{\mathbb{R}}^{{{{\mathop{\mathrm{D}}\nolimits} }_v}}}$ is the feature vector of node *i*$(i \in {{{\mathop{\mathrm{N}}\nolimits} }_v})$, and *N_v_* is the number of amino acid residues contained in a protein. ${{\bf A}} \in {{\mathbb{R}}^{{{{\mathrm{N}}}_v} \times {{{\mathrm{N}}}_v}}}$ is the adjacency matrix of the graph **G**, ${{\bf E}} \in \{ {{{{{\bf e}}}_{ij}}|{{{{\bf A}}}_{ij}} = 1} \}$ is the edge set, and **e***_ij_* is the feature vector of the edge between nodes *i* and *j*. That is, the elements of **E** are determined by the adjacency matrix **A**: if **A***_ij_* = 1, then ${{{{\bf e}}}_{ij}} \in {{\bf E}}$; if **A***_ij_* = 0, then ${{{{\bf e}}}_{ij}} \notin {{\bf E}}$.

#### Node representations

In protein graphs, the amino acid node features are derived from protein sequence and structure, and their extraction process follows the AGAT-PPIS [16]. All amino acid node feature vectors are combined together to form an amino acid node feature matrix ${{{{\bf X}}}_{\textit{node}}}$. Table 1 describe the features of these amino acids.

**Table 1:** Summary of amino acid node features

Features	Dimension	Category	Description
PSSM	20	Sequence information	Position-specific scoring matrix
HMM	20	Sequence information	Hidden Markov model matrix
DSSP	14	Structure information	Define secondary structure of proteins
AF	7	Structure information	Atomic features
PPE	1	Structure information	Pseudo-position embedding

##### Protein sequence features

The PSSM and the hidden Markov model matrix (HMM) are important features used to characterize the evolutionary information of protein sequences. The PSSM is generated using the PSI-BLAST v2.10.1 tool [29], while the HMM matrix is constructed using the HHblits v3.0.3 algorithm [30]. To further optimize the feature representation, the original values in the matrices are normalized to standardized scores ranging between 0 and 1, ultimately resulting in the feature matrices ${{{{\bf X}}}_{{\mathop{\mathrm{PSSM}}\nolimits} }} \in {{\mathbb{R}}^{{{{\mathop{\mathrm{N}}\nolimits} }_v} \times 20}}$and ${{{{\bf X}}}_{HMM}} \in {{\mathbb{R}}^{{{{\mathop{\mathrm{N}}\nolimits} }_v} \times 20}}$, respectively.

##### Protein structure features

Protein structural features include the following: define the secondary structure of proteins (DSSP) features, atomic features (AFs), and pseudo-position embedding (PPE) feature. DSSP features are generated using the DSSP algorithm [31], which is composed of 14-dimensional residue features. Specifically, the first 9 features are represented in one-hot encoding format to indicate the secondary structure states of the protein chain, covering the following secondary structure types: H (α-helix), G (310-helix), I (π-helix), E (extended strand), B (isolated bridge), T (turn), S (bend), and C (other or unknown secondary structures). The next 4 features are derived by applying sine and cosine transformations to the backbone torsion angles PHI and PSI of the peptide chain. Another feature is generated by converting the solvent accessible surface area into relative solvent accessibility. Ultimately, 14 features are combined together to form the structural feature matrix ${{{{\bf X}}}_{{\mathop{\mathrm{DSSP}}\nolimits} }} \in {{\mathbb{R}}^{{{{\mathop{\mathrm{N}}\nolimits} }_v} \times 14}}$ for representing 1 protein; here, *N_v_* is the number of amino acid residues contained in a protein.

AFs are the 7 attributes of each nonhydrogen atom in a residue, including atomic mass, B factor, whether it is a residue side chain atom, electron charge, the number of hydrogen atoms bonded to it, whether it is part of a ring, and the van der Waals radius of the atom. Since the number of atoms in each residue may vary, to standardize the feature representation, the average values of the 7 features for all atoms in each residue are calculated. Therefore, 7 features are combined together to form the atomic feature matrix ${{{{\bf X}}}_{{\mathop{\mathrm{AF}}\nolimits} }} \in {{\mathbb{R}}^{{{{\mathop{\mathrm{N}}\nolimits} }_v} \times 7}}$ for representing 1 protein.

The PPE feature of amino acid residues is used to characterize the relative positional information of each residue with respect to a reference residue. In this work, the coordinates of the side chain centroid (SC) are adopted as the pseudo-position representation of residues, thereby generating the pseudo-position embedding matrix ${{{{\bf X}}}_{{\mathop{\mathrm{PPE}}\nolimits} }} \in {{\mathbb{R}}^{{{{\mathop{\mathrm{N}}\nolimits} }_v} \times 1}}$.

The protein sequence feature matrices (i.e.,${{{{\bf X}}}_{{\mathop{\mathrm{PSSM}}\nolimits} }}$, ${{{{\bf X}}}_{HMM}}$) are concatenated with the structure feature matrices (i.e., ${{{{\bf X}}}_{{\mathop{\mathrm{DSSP}}\nolimits} }}$, ${{{{\bf X}}}_{{\mathop{\mathrm{AF}}\nolimits} }}$, ${{{{\bf X}}}_{{\mathop{\mathrm{PPE}}\nolimits} }}$) to form a unified feature matrix ${{{{\bf X}}}_{\textit{node}}} \in {{\mathbb{R}}^{{{{\mathrm{N}}}_v} \times 62}}$to represent 1 protein.


\begin{eqnarray*}
{{{{\bf X}}}_{\textit{node}}} = \left[ {{{{{\bf X}}}_{{\mathrm{PSSM}}}},{{{{\bf X}}}_{HMM}},{{{{\bf X}}}_{\textit{DSSP}}},{{{{\bf X}}}_{{\mathrm{AF}}}},{{{{\bf X}}}_{{\mathop{\mathrm{PPE}}\nolimits} }}} \right]
\end{eqnarray*}

#### Edge representations

Edge features in protein graphs focus on the spatial relationships between amino acid nodes. We extract the positional data of amino acids from the PDB file and calculate the Euclidean distance between them. By setting a distance threshold, we evaluate whether 2 amino acid nodes meet specific relationship criteria. If the distance is below the threshold, we create an edge between these 2 amino acid nodes. According to [16], we set the cutoff distance hyperparameter to 14 Å, and thus we can obtain an adjacency matrix **A** of a protein, where ${{{{\bf A}}}_{ij}} = 1$ indicates the presence of an edge between node *i* and node *j*, and ${{{{\bf A}}}_{ij}} = 0$ indicates no edge.

The computation of edge features involves 2 types of positional encoding: Euclidean distance between 2 nodes and the cosine value of the angle between 2 nodes. Thus, we can obtain the edge feature matrix ${{{{\bf X}}}_{\textit{edge}}} \in {{\mathbb{R}}^{{{{\mathrm{N}}}_e} \times 2}}$; here, *N_e_* represents the number of edges in a protein graph.

### MGMA-PPIS

**Figure 1:**
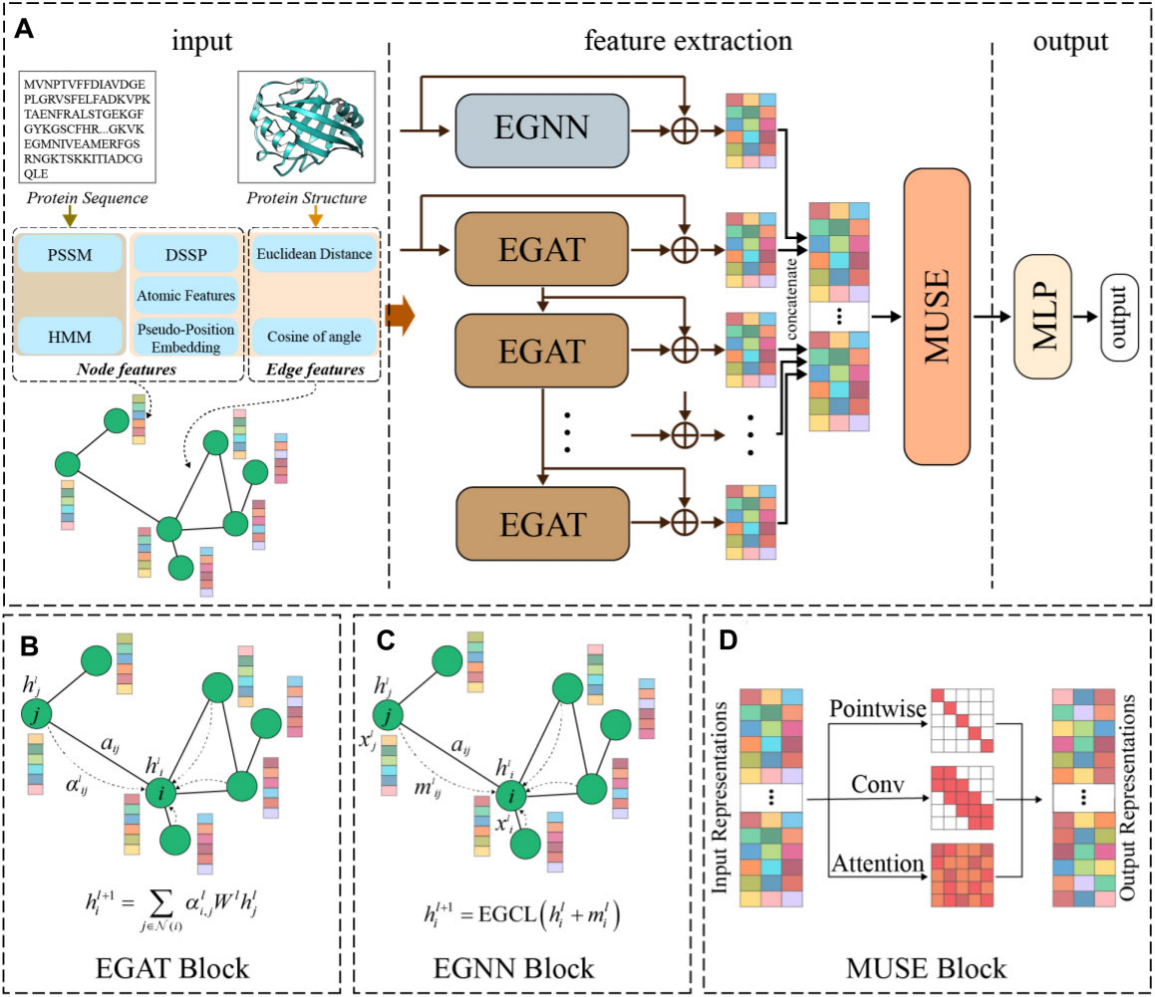
Overview of the MGMA-PPIS framework. (A) MGMA-PPIS consists of 3 principal components: input module, feature extraction module, and output module. In the input module, protein sequence and its structure are utilized to derive node features, edge features, and an adjacency matrix for building a protein graph with node/edge representation. In the feature extraction module, the protein graphs are processed using EGNN and EGAT to extract multiview graph features, which are integrated via the MUSE. In the output module, MLP is employed to further refine the node embeddings to output the prediction results of PPI sites. (B) EGAT Block, in which the protein graph is processed through EGAT with residual connections to extract multiscale local node features across various neighborhood levels. (C) EGNN Block, in which the protein graph is processed through EGNN with residual connections to capture the global node features. (D) MUSE Block, which integrates self-attention mechanisms, depth-wise separable convolutions, and position-wise feed-forward networks to enhance the feature representation.

In this work, we formulate the prediction of PPI binding sites as a graph node classification task and propose a feature fusion framework (named MGMA-PPIS) that combines multiview graph embeddings with a multiscale attention mechanism to predict PPI sites. The overall architecture of MGMA-PPIS is illustrated in Fig. 1. MGMA-PPIS consists of 3 main components: the input module, the feature extraction module, and the output module (Fig. 1A). First, MGMA-PPIS constructs the protein graph and extracts the node features and edge features from the protein sequence and structure, respectively. Subsequently, the protein graph with node and edge features is fed into the feature extraction module that consists of an E(n) EGNN [32] with residual connections and an EGAT [33] with residual connections. By concatenating the global node features extracted from the EGNN with the multiscale local node features extracted from the EGAT at different neighborhood scales through a multiscale attention network (MUSE), we can obtain the node embeddings of amino acids in the protein graph. In the end, these node embeddings are fed into an MLP to output the prediction results of PPI sites.

#### Edge graph attention network

The graph attention network (GAT) [34] extends GNNs [35] by not only defining local interactions through edges but also leveraging them to store temporary attention scores (i.e., edge weights) that quantify and adjust the importance of each connection. The initial idea of GAT is to compute independent attention scores for each edge in the graph, enabling GAT to learn which connections are crucial for information propagation between nodes and their neighbors. However, in GAT implementation, the intrinsic information of edges is neither adequately considered nor learned and cannot be effectively propagated through the network, thereby limiting the capacity of GAT for representation. To address this limitation, EGAT extends GAT by effectively incorporating edge features, enhancing its capability to model graph-structured data. EGAT updates the edge features ${{\bf e}}_{ij}^{l + 1}$ in an *l* + 1 layer using the node features ${{\bf h}}_i^l$ and edge features ${{\bf e}}_{ij}^l$ at layer *l*, then utilizes ${{\bf e}}_{ij}^{l + 1}$ to compute edge weights of the neighboring nodes, aggregating their node features to update the node features ${{\bf h}}_i^{l + 1}$. Consequently, EGAT can more efficiently learn the local information of protein from neighboring nodes based on the attention weights. As the number of layers increases, EGAT is capable of capturing the features from higher-order neighboring nodes, thus obtaining local information over a broader range. However, as the depth of a GNN increases, the oversmoothing phenomenon often occurs, causing the features of different nodes in the graph to become indistinguishable. To mitigate this issue, residual connections are introduced between EGAT layers to enhance the extraction of deep node features, thereby enabling more effective capture of key structural information of the protein graph. The update rules for node features and edge features are defined as follows:


\begin{eqnarray*}
{{\bf e}}_{ij}^{l + 1} = {\mathrm{LeakyReLU}}({{{{\bf A}}}^l}\left[ {{{\bf h}}_i^l\parallel {{\bf e}}_{ij}^l\parallel {{\bf h}}_j^l} \right]){\mathrm{, l = }}1,2, \cdot \cdot \cdot ,Z
\end{eqnarray*}

\begin{eqnarray*}
\alpha _{ij}^l = {\mathrm{Softmax}}({{\vec{F}}^l}({{\bf e}}_{ij}^{l + 1}))
\end{eqnarray*}

\begin{eqnarray*}
{{\bf h}}_i^{l + 1} = \textit{ReLU}({{\bf h}}_i^l + \sum\limits_{j \in \mathcal{N}(i)} {\alpha _{ij}^l{{{{\bf W}}}^l}{{\bf h}}_j^l} )
\end{eqnarray*}

where symbol ($\cdots \parallel \cdots $) denotes the vector concatenation operation, ${{\bf e}}_{ij}^{l + 1}$ is the updated edge feature, ${{\bf h}}_i^l$ is the features of node *i* in the *l* layer, ${{\bf h}}_j^l$ is the features of node *j* in the *l* layer, ${{\bf e}}_{ij}^l$ is the edge feature between nodes *i* and *j* in the *l* layer, **A** is a learnable matrix, and *LeakyReLU* is an activation function. The attention weight coefficient $\alpha _{ij}^{}$ between node *i* and node *j* is derived from the updated edge feature ${{\bf e}}_{ij}^{l + 1}$ using a learnable weight vector $\vec{F}$ and normalized via the Softmax function. The updated node feature ${{\bf h}}_i^{l + 1}$ is obtained by performing a weighted summation of the neighboring node features ${{\bf h}}_j^l$ based on the attention weight coefficients $\alpha _{ij}^{}$, as well as adding the result before updating ${{\bf h}}_i^l$, followed by a *ReLU* activation function.

In this work, we process the node feature matrix ${{{{\bf X}}}_{\textit{node}}}$ through an FC network to obtain the node embedding feature matrix ${{\bf X}}_{\textit{node}}^{{\mathrm{EGAT}}} = {{[ {{{\bf h}}_1^0,{{\bf h}}_2^0, \cdot \cdot \cdot ,{{\bf h}}_{{{{\mathrm{N}}}_v}}^0} ]}^{\mathrm{T}}}$, ${{\bf X}}_{\textit{node}}^{{\mathrm{EGAT}}} \in {{\mathbb{R}}^{{{{\mathrm{N}}}_v} \times 128}}$, and the node embedding feature matrix ${{\bf X}}_{\textit{node}}^{{\mathrm{EGAT}}}$ and the edge matrix ${{{{\bf X}}}_{\textit{edge}}}$ are used as the inputs of EGAT. Then, we treat the output ${{\bf h}}_i^l$ of EGAT at different layers as the local features of nodes and concatenate these features together to form the multiscale local feature ${{\bf h}}_i^{\textit{local}}$ of node *i*.


\begin{eqnarray*}
{{\bf h}}_i^{\textit{local}} = \left[ {{{\bf h}}_i^1\parallel {{\bf h}}_i^2\parallel \cdot \cdot \cdot \parallel {{\bf h}}_i^L} \right]
\end{eqnarray*}

where *L* is the total number of layers in EGAT, and symbol ($\cdots \parallel \cdots $) denotes the vector concatenation operation.

#### Equivariant graph neural network

EGNN introduces a novel architecture that is equivariant to translation, rotation, reflection (E(n)), and permutation, serving as a variant of GNN. Therefore, leveraging EGNN for protein feature extraction can better capture its structural properties. The network architecture is composed of a stack of equivariant graph convolutional layers (EGCLs). EGCL updates the node coordinate features ${{\bf x}}_i^{l + 1}$ and node features ${{\bf h}}_i^{l + 1}$ for the next layer by aggregating edge features ${{\bf e}}_{ij}^{}$ (between node *i* and node *j*) and combining the node coordinate features ${{\bf x}}_i^l$ and node features ${{\bf h}}_i^l$ from the current layer. The update rules for node coordinates and features are defined as follows:


\begin{eqnarray*}
{{\bf m}}_{ij}^l = {{\phi }_e}\left( {{{\bf h}}_i^l,{{\bf h}}_j^l,{{{\left\| {{{\bf x}}_i^l - {{\bf x}}_j^l} \right\|}}^2},{{{{\bf e}}}_{ij}}} \right)
\end{eqnarray*}

\begin{eqnarray*}
{{\bf x}}_i^{l + 1} = {{\bf x}}_i^l + C\sum\limits_{j \ne i} {({{\bf x}}_i^l + {{\bf x}}_j^l){{\phi }_x}({{\bf m}}_{ij}^l)}
\end{eqnarray*}

\begin{eqnarray*}
{{\bf m}}_i^l = \sum\limits_{j \ne i} {({{\bf m}}_{ij}^l)}
\end{eqnarray*}

\begin{eqnarray*}
{{\bf h}}_i^{l + 1} = \textit{ReLU}({{\bf h}}_i^l + {{\phi }_h}({{\bf h}}_i^l + {{\bf m}}_i^l))
\end{eqnarray*}

where ${{\bf m}}_{ij}^l$ represents the edge embedding between node *i* and node *j* at layer *l*, ${{\phi }_e}$ is an edge operation, ${{\phi }_x}$ is a coordinate operation, $C = {1 / {(M - 1)}}$ is a constant factor, *M* is the total number of nodes in the graph, and ${{\phi }_h}$ is a node operation.

When updating coordinates and node features, EGNN integrates the node coordinates, node information, and edge information of the entire graph to capture more global information. In contrast to EGAT, which primarily captures the local information, EGNN is more adept at capturing the global information. Therefore, we use EGNN to extract the global features of proteins.

Similarly, we process the node feature matrix ${{{{\bf X}}}_{\textit{node}}}$ through an FC network to generate the node embedding feature matrix ${{\bf X}}_{\textit{node}}^{{\mathrm{EGNN}}} = {{[ {{{\bf h}}_1^0,{{\bf h}}_2^0, \cdot \cdot \cdot ,{{\bf h}}_{{{{\mathrm{N}}}_v}}^0} ]}^{\mathrm{T}}}$, ${{\bf X}}_{\textit{node}}^{{\mathrm{EGNN}}} \in {{\mathbb{R}}^{{{{\mathrm{N}}}_v} \times 256}}$, then input the node embedding feature matrix ${{\bf X}}_{\textit{node}}^{{\mathrm{EGNN}}}$ and edge matrix ${{{{\bf X}}}_{\textit{edge}}}$ into EGNN. The output ${{\bf h}}_i^{\textit{global}} = {{\bf h}}_i^{\mathrm{Y}}$ of the last layer is taken as the global features of nodes, where *Y* is the number of layers in EGNN. Finally, we concatenate the global feature ${{\bf h}}_i^{\textit{global}}$ extracted by EGNN with the multiscale local features ${{\bf h}}_i^{\textit{local}}$ extracted by EGAT to generate the final node feature vector ${{\bf h}}_i^{all}$ of node *i*, ${{\bf h}}_i^{all} = [ {{{\bf h}}_i^{\textit{global}}\parallel {{\bf h}}_i^{\textit{local}}} ]$. Thereby, we obtain the multiview feature embedding matrix ${{{{\bf X}}}_{\textit{embedding}}}$ of 1 protein, ${{{{\bf X}}}_{\textit{embedding}}} = {{[ {{{\bf h}}_1^{all},{{\bf h}}_2^{all}, \cdot \cdot \cdot ,{{\bf h}}_{{{{\mathrm{N}}}_v}}^{all}} ]}^{\mathrm{T}}}$.

#### Multiscale attention network

To focus on important features, we introduce the attention-based feature representation learning to process protein sequences. In sequence learning, the self-attention mechanism has proven to be highly efficient and has achieved significant performance improvements across numerous tasks [36, 37]. However, the attention in deep layers often overly focuses on a single token, which not only limits the full utilization of local information but also shows inadequacies when representing long sequences. Therefore, to more effectively capture both long-range and short-range linguistic structures, the multiscale attention mechanism with parallelization is proposed for parallel multiscale representation learning on sequential data [27]. Therefore, we integrate convolution operations and self-attention mechanisms to encode protein sequences in parallel across multiple scales, embodying the core principle of parallel multiscale sequence representation learning. Our MUSE block consists of 3 main components: self-attention for capturing the global contextual features, depth-wise separable convolution for extracting the local patterns, and position-wise feed-forward network for capturing the token features.

By leveraging the characteristics of MUSE in sequence representation learning, we adopt MUSE to the multiview feature embedding matrix ${{{{\bf X}}}_{\textit{embedding}}}$ and use a fusion trick to output a representation ${{{{\bf X}}}_{\textit{muse}}}$.


\begin{eqnarray*}
{{{{\bf X}}}_{\textit{muse}}} &=&{{{{\bf X}}}_{\textit{embedding}}}{\mathrm{ + Attention}}({{{{\bf X}}}_{\textit{embedding}}}) + {\mathrm{Conv}}({{{{\bf X}}}_{\textit{embedding}}})\\
&& + {\mathrm{Pointwise}}({{{{\bf X}}}_{\textit{embedding}}})
\end{eqnarray*}

where “Attention” is the self-attention operation, “Conv” is the depth-wise separable convolution operation, and “Pointwise” is the position-wise feed-forward operation.

The self-attention responsible for learning global contextual representations projects the multiscale feature matrix ${{{{\bf X}}}_{\textit{embedding}}}$ into 3 distinct representations: key *K*, query *Q*, and value *V*, for computing the output representation.


\begin{eqnarray*}
{\mathrm{Attention}}({{\bf X}}){\mathrm{ = }}\sigma \left( {{{\bf Q}},{{\bf K}},{{\bf V}}} \right){{W}^O}
\end{eqnarray*}

\begin{eqnarray*}
\sigma \left( {{{\bf Q}},{{\bf K}},{{\bf V}}} \right) = {\mathrm{softmax}}\left( {{{{{\bf Q}}{{{{\bf K}}}^{\mathrm{T}}}} / {\sqrt {{{d}_k}} }}} \right){{\bf V}}
\end{eqnarray*}

\begin{eqnarray*}
{{\bf Q}} = {{{{\bf X}}}_{\textit{embedding}}}{{W}^Q},\ {{\bf K}} = {{{{\bf X}}}_{\textit{embedding}}}{{W}^K},\ {{\bf V}} = {{{{\bf X}}}_{\textit{embedding}}}{{W}^V}
\end{eqnarray*}

where ${{W}^Q}$, ${{W}^K}$, ${{W}^V}$, and ${{W}^O}$ are the projection parameters, and $\sigma ( \cdots )$ is the self-attention operation, which is defined as the dot product between the key, query, and value pairs.

The depth-wise separable convolution as the convolutional component (including depth-wise convolution and point-wise convolution) within a parallel structure can effectively compensate for the insufficient utilization of local information, while the self-attention mechanism focuses on capturing global dependencies [38]. Each convolutional submodule contains multiple units with different kernel sizes to capture features at various scales. This was introduced in [39]. For an input sequence **X**, the computation of output **O** is formulated as follows:


\begin{eqnarray*}
{{{{\bf O}}}_{i,c}} = {\mathrm{DepthCon}}{{{\mathrm{v}}}_k}({{\bf X}}) = \sum\limits_{j = 1}^k {{{W}_{c,j}} \cdot {{{{\bf X}}}_{(i + j - \left\lceil {{{(k + 1)} / 2}} \right\rceil ),c}}}
\end{eqnarray*}

where “DepthConv” is the depth-wise separable convolution operation, and *k* is the kernel width.

The formulation of the point-wise separable convolution with a kernel size of *k* is as follows:


\begin{eqnarray*}
{\mathrm{Con}}{{{\mathrm{v}}}_k}({{\bf X}}) = {\mathrm{DepthCon}}{{{\mathrm{v}}}_k}({{{{\bf V}}}_2}){{W}^{out}}
\end{eqnarray*}

\begin{eqnarray*}
{{{{\bf V}}}_2} = {{{{\bf X}}}_{\textit{embedding}}}{{W}^V}
\end{eqnarray*}

where ${{W}^V}$ and ${{W}^{out}}$ are the projection parameters, and ${{W}^V}$ is a point-wise projecting matrix.

By adopting shared projections, the input features can be mapped into the same hidden space, enabling better learning of contextual sequence representations [40]. Therefore, the projection operations in both the self-attention mechanism ${{\bf V}} = {{{{\bf X}}}_{\textit{embedding}}}{{W}^V}$ and the convolution mechanism ${{{{\bf V}}}_2} = {{{{\bf X}}}_{\textit{embedding}}}{{W}^V}$ are shared.

In addition, dynamic convolution (the optimal variant of DepthConv) is employed in this work. Due to automatically assigning the convolution kernels, we apply the following formula to normalize the weights:


\begin{eqnarray*}
{\mathrm{Conv}}({{\bf X}}) = \sum\limits_{i = 1}^n {\frac{{\exp ({{\alpha }_i})}}{{\sum\limits_{j = 1}^n {\exp ({{\alpha }_j})} }}} {\mathrm{Con}}{{{\mathrm{v}}}_{{{k}_i}}}({{\bf X}})
\end{eqnarray*}

where α*_i_* denotes the weight of DepthConv output network.

The position-wise feed-forward network is used to capture token representations. To learn token-level feature representations, the parallel structure incorporates a position-wise feed-forward network. Since the linear transformation is identical across different positions, it can be regarded as a token feature extractor for capturing the feature representation of each token.


\begin{eqnarray*}
{\mathrm{Pointwise}}({{\bf X}}) = \textit{ReLu}({{\bf X}}{{W}_1} + {{b}_1}){{W}_2} + {{b}_2}
\end{eqnarray*}

where ${{W}_1}$, ${{b}_1}$  ${{W}_2}$, and ${{b}_2}$ are the projection parameters of the feed-forward neural network.

### Focal loss

Cross-entropy loss (CE) is a traditional loss function that is widely used for binary classification tasks. However, the datasets used in this work exhibit a highly imbalanced class distribution (as shown in Supplementary Table S1). Such imbalance typically leads to a strong bias, causing the model to favor the majority class. When training a model using the cross-entropy loss function, the optimization objective is to minimize the average loss over the entire training dataset. As a result, model tends to perform well on majority class samples but poorly on minority class samples. Therefore, in this work, we adopt focal loss (FL) as the loss function to mitigate the adverse effects of class imbalance on model performance. FL not only assigns asymmetric weights to samples from different classes but also differentiates between easy and hard examples by applying different weights, which helps reduce the bias introduced during training. Focal loss is derived from the CE:


\begin{eqnarray*}
CE(p,y) = \left\{ \begin{array}{@{}l@{}} \ - \log (p)\ if\ y = 1\\ \\ \\ \ - \log (1 - p)\ \textit{otherwise} \end{array} \right.
\end{eqnarray*}

where $y \in \{ { { \pm 1} \}} $ denotes the ground truth for negative and positive classes, respectively, and $p \in [ {0,1} ]$ represents the estimated probability for the class with label *y* = 1.

By incorporating a modulating factor ${{(1 - {{p}_t})}^\gamma }$ into the CE with an adjustable focusing parameter $\gamma \ge 0$, we reduce the weighting of easy examples to mitigate the influence of simple examples, thereby shifting the training focus toward challenging negatives. Thus, focal loss can be formulated as


\begin{eqnarray*}
{\mathrm{FL}}({{p}_t}) = - {{(1 - {{p}_t})}^\gamma }{\mathrm{CE}}({{p}_t}) = - \alpha {{(1 - {{p}_t})}^\gamma }\log ({{p}_t})
\end{eqnarray*}

\begin{eqnarray*}
{{p}_t} = \left\{ \begin{array}{@{}l@{}} \ p\ if\ y = 1\\ \\ \\ \ 1 - p\ \textit{otherwise} \end{array} \right.
\end{eqnarray*}

where $\alpha $ is a weighting factor, which is used to address the imbalance between positive and negative samples. Focal loss enables the classifier to primarily focus on minority class samples and hard-to-classify examples.

### Evaluation metrics

In this work, we use accuracy (ACC), precision, recall, F1-score (F1), Matthews correlation coefficient (MCC), area under the receiver operating characteristic curve (AUROC), and area under the precision-recall curve (AUPRC) to evaluate the model performance. The formulas of ACC, precision, recall, F1, and MCC are as follows:


\begin{eqnarray*}
{\mathop{\mathrm{ACC}}\nolimits} = \frac{{TP + TN}}{{TP + TN + FP + FN}}
\end{eqnarray*}

\begin{eqnarray*}
{\mathrm{Precision}} = \frac{{TP}}{{TP + FP}}
\end{eqnarray*}

\begin{eqnarray*}
{\mathrm{Recall}} = \frac{{TP}}{{TP + FN}}
\end{eqnarray*}

\begin{eqnarray*}
{\mathop{\mathrm{F}}\nolimits} 1 = \frac{{2{\mathrm{ \times }}{\mathop{\mathrm{Precision}}\nolimits} {\mathrm{ \times }}{\mathop{\mathrm{Recall}}\nolimits} }}{{{\mathop{\mathrm{Precision}}\nolimits} + {\mathop{\mathrm{Recall}}\nolimits} }}
\end{eqnarray*}

\begin{eqnarray*}
{\mathrm{MCC}} = \frac{{TP \times TN - FP \times FN}}{{\sqrt {(TP + FP)(TP + FN)(TN + FP)(TN + FN)} }}
\end{eqnarray*}

where TP and TN represent the number of correctly predicted interaction sites and noninteraction sites, respectively; FP and FN represent the number of incorrectly predicted interaction sites and noninteraction sites, respectively. AUROC and AUPRC are threshold-independent metrics that reflect model overall performance. Given the significant class imbalance between positive and negative samples in our work, we place particular emphasis on 3 evaluation metrics that are especially important for imbalanced datasets: F1, MCC, and AUPRC, in order to more accurately assess the model performance.

## Results and Discussion

In this work, MGMA-PPIS is implemented using Python 3.9 with PyTorch 2.3.0 and the Deep Graph Library (DGL) 2.4.0 packages. The primary criterion for performance evaluation is the average AUPRC obtained through the 5-fold cross-validation (CV) test, which is used to guide the selection and optimization of relevant features and hyperparameters. For the 5CV test, we take each protein as 1 sample and then randomly partition all proteins into 5 subsets with roughly equal size. One of the 5 subsets is singled out, in turn, as the testing set; 80% and 20% of the samples of the other 4 subsets are used as the training samples (forming training set) and validation samples (forming validation set), respectively. In the training set, validation set, and testing set, the interaction sites on each protein chain are considered positive samples, while other noninteraction sites are considered negative samples. Through experimental validation, we determine the final hyperparameter configurations as follows: 5 layers for EGAT, 7 layers for EGNN, and $\alpha $ = 0.25. Additionally, according to the insights from previous studies [23] and empirical validation, we set $\gamma $ = 2. The detailed information is presented in Supplementary Tables S2 and S3.

### Performance comparison of MGMA-PPIS with other methods

To comprehensively evaluate the performance of MGMA-PPIS, we first compare our MGMA-PPIS with other existing methods on an independent test dataset (i.e., Test_60). As shown in Table 2, MGMA-PPIS outperforms all other methods across the 7 metrics. Specifically, when comparing the graph convolution–based methods (i.e., AGAT-PPIS, AGF-PPIS, and GHGPR-PPIS) with the best prediction results among all comparison methods, our MGMA-PPIS increases ACC by 0.023–0.027, precision by 0.072–0.084, recall by 0.032–0.049, F1 by 0.053–0.088, MCC by 0.066–0.083, AUROC by 0.031–0.034, and AUPRC by 0.074–0.099. These results demonstrate the effectiveness and superiority of MGMA-PPIS in predicting PPI sites. In addition, the AUROC and AUPRC curves of MGMA-PPIS, AGAT-PPIS, and GHGRP-PPIS are shown in Supplementary Figs. S1 and S2.

**Table 2:** Result of MGMA-PPIS and other thirteen comparative methods on Test_60 dataset.

Method	ACC	Precision	Recall	F1	MCC	AUROC	AUPRC
PSIVER	0.561	0.188	0.534	0.278	0.074	0.573	0.190
ProNA2020	0.738	0.275	0.402	0.326	0.176	N/A	N/A
SCRIBER	0.667	0.253	0.568	0.350	0.193	0.665	0.278
DLPred	0.682	0.264	0.565	0.360	0.208	0.677	0.294
DELPHI	0.697	0.276	0.568	0.372	0.225	0.699	0.319
DeepPPISP	0.657	0.243	0.539	0.335	0.167	0.653	0.276
SPPIDER	0.752	0.331	0.557	0.415	0.285	0.755	0.373
MaSIF-site	0.780	0.370	0.561	0.446	0.326	0.775	0.439
GraphPPIS	0.776	0.368	0.584	0.451	0.333	0.786	0.429
DeepProSite	0.842	0.501	0.443	0.470	0.379	0.813	0.490
AGAT-PPIS	0.856	0.539	0.603	0.569	0.484	0.867	0.574
AGF-PPIS	0.860	0.551	0.620	0.584	0.501	0.870	0.599
GHGPR-PPIS	0.860	0.551	0.620	0.583	0.501	0.869	0.596
MGMA-PPIS	**0.883**	**0.623**	**0.652**	**0.637**	**0.567**	**0.901**	**0.673**

Considering that the performance of AGAT-PPIS, AGF-PPIS, and GHGPR-PPIS on Test_60 dataset is significantly better than the other 10 comparison methods, we compare our MGMA-PPIS with these 3 methods on the other 3 independent test sets (i.e., Test_315–28, Btest_31–6, and UBtest_31–6) to further validate the performance of MGMA-PPIS. As shown in Table 3 (see footnote; [24]), we can see that the MCC and AUPRC metrics of our MGMA-PPIS are still higher than that of the other 3 methods, indicating that our MGMA-PPIS has superior generalization ability.

**Table 3:** Results of MGMA-PPIS and other the 3 comparison methods on Test_315–28, BTest_31–6, and UBtest_31–6 datasets

	Test_315–28		Btest_31–6		UBtest_31–6	
Method	MCC	AUPRC	MCC	AUPRC	MCC	AUPRC
AGAT-PPIS	0.481	0.572	0.485	0.583	0.327	0.365
AGF-PPIS	0.484	0.565	0.518	0.604	0.339	0.370
GHGPR-PPIS	0.486	0.566	N/A	N/A	0.356	0.367
MGMA-PPIS	**0.535**	**0.623**	**0.540**	**0.604**	**0.374**	**0.404**

^∗^N/A indicates that the corresponding value is not available in the original literature.

The above results demonstrate that our multiview graph embedding and multiscale attention-based feature fusion consistently outperform other competitive methods. The superiority of our MGMA-PPIS stems from a novel design framework in which EGAT extracts local features across multiple neighborhood scales, EGNN captures global structural features, and a multiscale attention mechanism adaptively emphasizes the most discriminative components. In contrast, other methods [16, 23] typically employ a single graph neural network without distinguishing local and global features, and they rely on simple concatenation or conventional self-attention for fusion. The methodological innovations and structural differences between our MGMA-PPIS and other methods are illustrated in Supplementary Fig. S3.

### Ablation experiments of diverse architecture components in MGMA-PPIS

In the MGMA-PPIS framework, EGAT is responsible for extracting local features from different neighborhoods within the protein graph, EGNN captures the global features of the protein graph, and the multiscale attention network serves as the feature fusion mechanism. To investigate the contributions of these key components for improving MGMA-PPIS performance, we design 3 variants (i.e., MGMA-PPIS_-EGAT_, MGMA-PPIS_-EGNN_, and MGMA-PPIS_-MUSE_) of MGMA-PPIS to compare MGMA-PPIS and its 3 variants on the training dataset in terms of average AUROC and AUPRC using the 5-fold cross-validation test, as well as compare MGMA-PPIS and its 3 variants on the independent Test_60 dataset in terms of AUROC and AUPRC. MGMA-PPIS_-EGAT_ represents using the traditional GCN instead of EGAT in the MGMA-PPIS framework. MGMA-PPIS_-EGNN_ represents using the traditional GCN instead of EGNN in the MGMA-PPIS framework. MGMA-PPIS_-MUSE_ represents removing the multiscale attention network from the MGMA-PPIS framework but using the same graph neural network architecture.

Table 4 presents the performance comparison between MGMA-PPIS and its 3 variants. The results clearly show that MGMA-PPIS outperforms all variants in both AUROC and AUPRC, underscoring the effectiveness of each proposed component. In particular, EGNN, which is equivariant to translation, rotation, reflection (E(n)), and permutation, demonstrates superior capability in capturing the global features of protein graphs compared to conventional GNNs. Likewise, EGAT, which effectively incorporates edge features, exhibits enhanced ability in extracting local features from different neighborhoods of protein graphs. Furthermore, the multiscale attention network serves as an advanced feature fusion strategy, delivering notable performance improvements over basic feature concatenation. Overall, these 3 components contribute significantly to the overall performance of MGMA-PPIS, with EGNN providing the greatest impact.

**Table 4:** Results of MGMA-PPIS and its 3 variants on the validation and Test_60 datasets

	Validation dataset		Test_60 dataset	
Method	AUROC	AUPRC	AUROC	AUPRC
MGMA-PPIS_-MUSE_	0.878	0.617	0.884	0.640
MGMA-PPIS_-EGAT_	0.886	0.649	0.883	0.607
MGMA-PPIS_-EGNN_	0.820	0.495	0.825	0.504
MGMA-PPIS	**0.887**	**0.648**	**0.901**	**0.673**

### Effectiveness of protein structure features

In our MGMA-PPIS, we extract the protein sequence features and its structure features. The sequence features are derived from PSSM and HMM, while the structural features include DSSP, AF, and PPE. Given that the importance of PSSM, HMM, and DSSP has been extensively validated in previous studies [16], we do not reevaluate these features. Here we investigate the impact of protein structure features (i.e., AF and PPE) on MGMA-PPIS by combining the sequence features (i.e., HMM + PSSM + DSSP) with AF and PPE structure features. The results of MGMA-PPIS with different structure features are shown in Table 5, from which we can see that protein structure features (i.e., AF and PPE) can effectively improve the performance of PPI site prediction. For example, the AUROC and AUPRC of MGMA-PPIS with HMM + PSSM + DSSP + AF combination features on Test_60 dataset are 0.042, 0.094 higher than that of HMM + PSSM + DSSP combination features, respectively. The AUROC and AUPRC of MGMA-PPIS with HMM + PSSM + DSSP + AF + PPE combination features on the Test_60 dataset are 0.011, 0.026 higher than that of HMM + PSSM + DSSP + AF combination features, respectively. Thus, in this work, we adopt the combination features of HMM + PSSM + DSSP + AF + PPE in the MGMA-PPIS framework to predict PPI sites.

**Table 5:** Results of MGMA-PPIS with different combination features on the validation and Test_60 datasets

	Validation dataset		Test_60 dataset	
Combination features	AUROC	AUPRC	AUROC	AUPRC
HMM + PSSM + DSSP	0.833	0.520	0.848	0.553
HMM + PSSM + DSSP + AF	0.885	0.641	0.890	0.647
HMM + PSSM + DSSP + AF + PPE	**0.887**	**0.648**	**0.901**	**0.673**

### Effectiveness of the multiscale attention network

**Figure 2:**
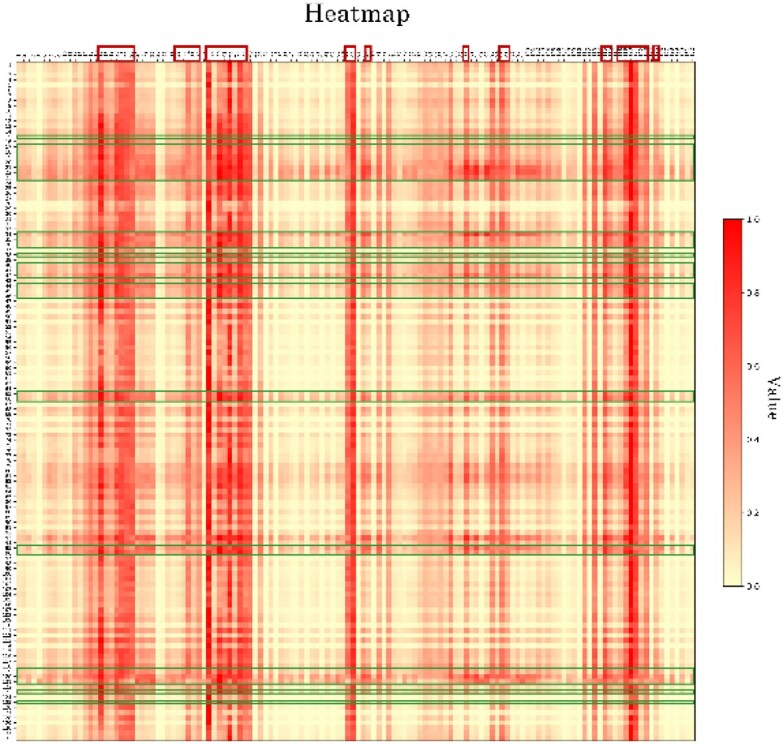
Attention weight heatmap generated by MGMA-PPIS for chain A in the 2yc2 protein.

To rigorously assess whether applying the MUSE for feature embedding fusion can more effectively capture key information, we conducted a comparative study using both the traditional self-attention network and multiscale attention network for feature fusion in protein sequence processing, denoted as methods MGMA-PPIS_-SA_ and MGMA-PPIS, respectively. Their performances were evaluated on the validation dataset and the independent Test_60 dataset. As presented in Table 6, MGMA-PPIS, equipped with the multiscale attention network, demonstrated superior performance in both the AUROC and AUPRC metrics. Moreover, we further analyzed the attention regions of the self-attention mechanism in MUSE when capturing global contextual features and visualized the prediction results (Fig. 2) for chain A in the 2yc2 protein. The results show that the attention weights of the predicted binding sites (outlined in green) are primarily concentrated on positions that correspond exactly to the actual binding sites (outlined in red).

**Table 6:** Results of MGMA-PPIS with different attention network on the validation and Test_60 datasets

	Validation dataset		Test_60 dataset	
Method	AUROC	AUPRC	AUROC	AUPRC
MGMA-PPIS_-SA_	0.882	0.631	0.887	0.644
MGMA-PPIS	**0.887**	**0.648**	**0.901**	**0.673**

### Effectiveness of different loss functions

To mitigate the impact of imbalanced positive and negative sample distributions on MGMA-PPIS training, we employ the focal loss instead of the traditional cross-entropy loss, then compare the performance of MGMA-PPIS with these 2 loss functions on both the validation dataset and the independent test dataset of Test_60. The experimental results are shown in Table 7, from which we can see that the performance of MGMA-PPIS with focal loss function is superior to that with the cross-entropy loss function. For example, the AUROC and AUPRC of MGMA-PPIS with focal loss function on the validation dataset are 0.002, 0.008 higher than those with the cross-entropy loss function, respectively. These results indicate that focal loss function can mitigate the impact of sample imbalance on model training.

**Table 7:** Results of MGMA-PPIS with different loss functions on the validation and Test_60 datasets

	Validation dataset		Test_60 dataset	
Loss function	AUROC	AUPRC	AUROC	AUPRC
Cross-entropy loss	0.885	0.640	0.896	0.660
Focal loss	0.887	0.648	0.901	0.673

### Running time analysis

**Figure 3:**
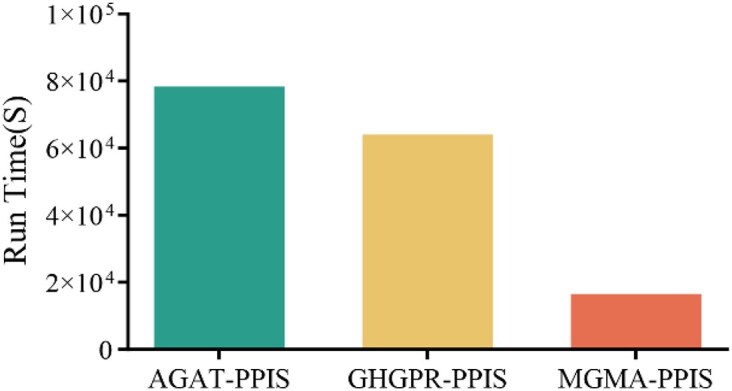
The running time of AGAT-PPIS, GHGPR-PPIS, and MGMA-PPIS on the validation dataset.

We compare the running time of our MGMA-PPIS with AGAT-PPIS and GHGPR-PPIS methods on the validation dataset. These 2 comparison methods have the best prediction results among all comparison methods. As shown in Fig. 3, we can see that the running time of our MGMA-PPIS is far lower than that of AGAT-PPIS and GHGPR-PPIS methods, indicating that our MGMA-PPIS has excellent computational efficiency.

### Case study

**Figure 4:**
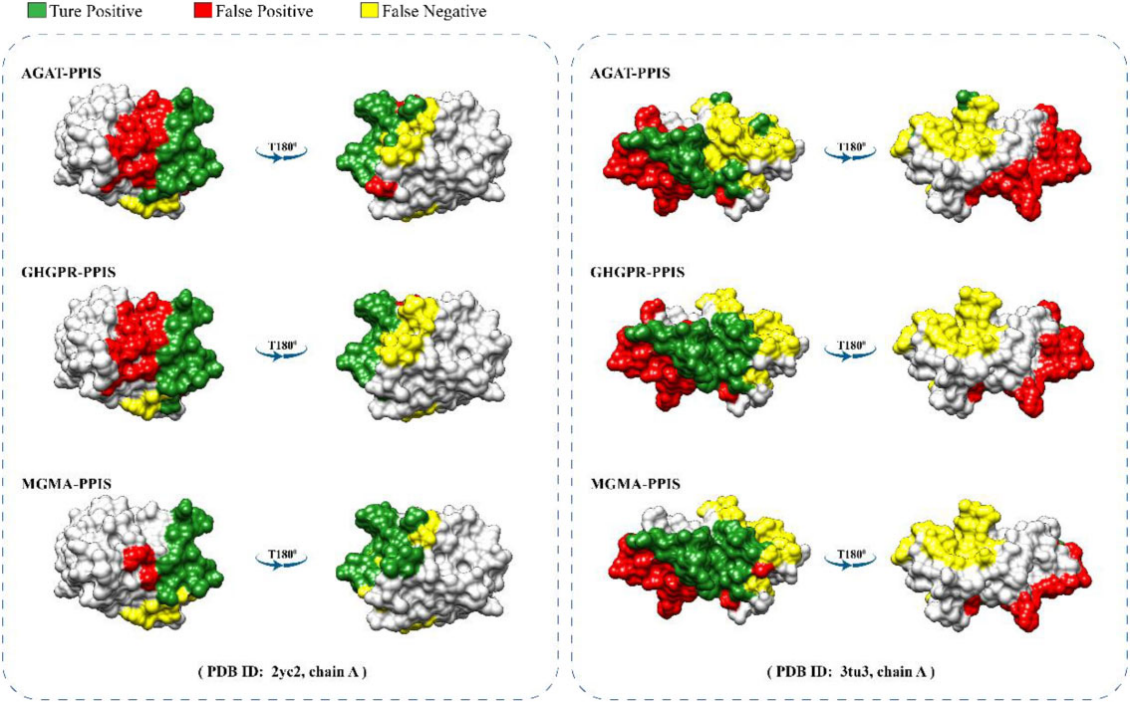
Visualization of prediction results of AGAT-PPIS, GHGPR-PPIS, and MGMA-PPIS on protein 2yc2 chain A and protein 3tu3 chain A.

Through the study of specific protein samples, we aim to further demonstrate the outstanding capability of the MGMA-PPIS in PPI site prediction. Table 8 and Fig. 4 present 2 case study results (i.e., PDB ID: 2yc2, chain A; PDB ID: 3tu3, chain A) of MGMA-PPIS with AGAT-PPIS and GHGPR-PPIS methods from the Test_60 dataset. As shown in Table 8 and Fig. 4, we can see that our MGMA-PPIS significantly outperforms AGAT-PPIS and GHGPR-PPIS. More protein prediction results are presented in Supplementary Table S4 and Supplementary Fig. S4. These results show that MGMA-PPIS can effectively reduce the number of false-positive sites, thereby improving the performance of PPI site prediction.

**Table 8:** Results of MGMA-PPIS, GHGPR-PPIS, and AGAT-PPIS on specific proteins (PDB ID: 2yc2, chain A and PDB ID: 3tu3, chain A)

PDB ID	Methods	TP	TN	FP	FN
2yc2, chain A	AGAT-PPIS	24	82	15	11
	GHGPR-PPIS	22	83	14	13
	MGMA-PPIS	25	95	2	10
3tu3, chain A	AGAT-PPIS	20	48	28	23
	GHGPR-PPIS	23	55	21	20
	MGMA-PPIS	24	59	17	19

## Conclusion

In this work, we develop an innovative MGMA-PPIS method for high-precision prediction PPI sites.

MGMA-PPIS integrates global node features extracted with EGNN and multiscale local node features extracted with EGAT at different neighborhood scales, thereby constructing a multiview graph feature representation, and then employs a multiscale attention network (MUSE) to deeply fuse these features across multiple scales, enabling the precise construction of embedding representations for amino acid nodes within protein graphs. Through a series of comparative experiments, we demonstrate that MGMA-PPIS outperforms other existing methods and exhibits superior generalization ability across all independent test sets.

Although our MGMA-PPIS has good predictive performance, it has the following limitation. As a structure-based method, MGMA-PPIS requires protein tertiary structure information, while the number of proteins with known tertiary structures is relatively limited, which to some extent restricts the MGMA-PPIS applicability. Looking ahead, future work may explore the integration of protein sequence and structure pretrained models into the feature extraction process to enrich and diversify the extracted features, thereby further enhancing the performance of PPI site prediction. Moreover, the architecture and methodology proposed in this work are not limited to the prediction of PPI sites but can also be extended to predict other types of protein binding sites, such as DNA-binding sites and drug-binding sites, offering new ideas and approaches for related research fields.

## Availability of Source Code and Requirements

Project name: MGMA-PPIS

Project homepage: https://github.com/NWPU-903PR/MGMA-PPIS

Operating system(s): Linux

Programming language: Python

Other requirements: NVIDIA GPU with CUDA 11.8 or higher

License: MGMA-PPIS codebase is licensed with a CC0 1.0 license (dataset) and the MIT license.

## Data Availability

The benchmark dataset was obtained from the GitHub repository [41].

## Additional Files

**Supplementary Fig. S1**. ROC curves of MGMA and the other two comparison methods on the Test_60 dataset.

**Supplementary Fig. S2**. PR curves of MGMA and the other two comparison methods on the Test_60 dataset.

**Supplementary Fig. S3**. Diagrammatic sketch of framework comparison between our MGMA-PPIS (right) with other existing methods (left). Unlike the single-view GNNs that employ simple feature concatenation or standard self-attention mechanisms, MGMA-PPIS extracts complementary local features across multiple neighborhood scales through EGAT, integrates global structural features via EGNN, and achieves multiview and multiscale information fusion using an multiscale attention mechanism.

**Supplementary Fig. S4**. Visualization of prediction results of AGAT-PPIS, GHGPR-PPIS, and MVMA-PPIS on specific protein samples.

**Supplementary Table S1**. The statistical information of 4 datasets.

**Supplementary Table S2**. Results of MGMA with different layer numbers on verification and Test_60 datasets.

**Supplementary Table S3**. Results of MGMA with different α values on verification and Test_60 datasets.

**Supplementary Table S4**. Results of MVMA-PPIS, GHGPR-PPIS, and AGAT-PPIS on a specific protein (PDB ID: 3q87, chain A and PDB ID: 2v9t, chain B).

giaf114_Supplemental_File

giaf114_Authors_Response_To_Reviewer_Comments_Original_Submission

giaf114_GIGA-D-25-00221_Original_Submission

giaf114_GIGA-D-25-00221_Revision_1

giaf114_Reviewer_1_Report_Original_Submission Juntao Liu -- 7/26/2025

giaf114_Reviewer_1_Report_Revision_1 Juntao Liu -- 8/18/2025

giaf114_Reviewer_2_Report_Original_Submission Jianmin Wang -- 7/28/2025

giaf114_Reviewer_2_Report_Revision_1 Jianmin Wang -- 8/24/2025

## Abbreviations

ACC: accuracy; AF: atomic feature; AUPRC: area under the precision-recall curve; AUROC: area under the receiver operating characteristic curve; CE: cross-entropy loss; CNN: convolutional neural network; CV: cross-validation; DSSP: define the secondary structure of proteins; EGAT: edge graph attention network; EGCL: equivariant graph convolutional layer; EGNN: equivariant graph neural network; FC: fully connected; FL: focal loss; GAT: graph attention network; GCN: graph convolutional neural network; GNN: graph neural network; HMM: hidden Markov model matrix; HSP: high-scoring segment pair; MCC: Matthews correlation coefficient; MLP: multilayer perceptron; MUSE: multiscale attention network; PPE: pseudo-position embedding feature; PPI: protein–protein interaction; PSSM: position-specific scoring matrix; RNN: recurrent neural network; SC: side chain centroid; SLSTM: simplified long short-term memory.

## Competing Interests

The authors declare that they have no competing interests.
