# Supplementary material for: MGMA-PPIS: Predicting the protein–protein interaction site with multiview graph embedding and multiscale attention fusion
Source: Gigascience. 2025 Oct 1;14:giaf114. doi: 10.1093/gigascience/giaf114 (PMC12486388; doi:10.1093/gigascience/giaf114)
Supplement: giaf114_Supplemental_File [file giaf114_supplemental_file.docx]

Supplementary Tables and Figures

Table S1. The statistical information of four datasets

| **Dataset** | **Protein chains** | **Abnormal Protein Chains** | **Interacting residues** | **Non-interacting residues** | **Proportion of interaction**  **site (%)** |
| --- | --- | --- | --- | --- | --- |
| Train_335-1 | 334 | 1 | 10336 | 55872 | 15.6 |
| Test_60 | 60 | 0 | 2075 | 11069 | 15.8 |
| Test_315-28 | 287 | 28 | 8566 | 51810 | 14.2 |
| Ubtest_31-6 | 25 | 6 | 711 | 5206 | 12.0 |

Table S2. Results of MGMA with different layer numbers on verification and Test_60 datasets

| Number in EGAT-EGNN layer | Verification dataset | | Test_60 dataset | |
| --- | --- | --- | --- | --- |
|  | AUROC | AUPRC | AUROC | AUPRC |
| 1-1 | 0.8524 | 0.5563 | 0.8581 | 0.5524 |
| 2-2 | 0.8672 | 0.5989 | 0.8771 | 0.6148 |
| 3-3 | 0.8804 | 0.6241 | 0.8615 | 0.5605 |
| 4-4 | 0.8805 | 0.6272 | 0.8810 | 0.6175 |
| 5-5 | 0.8830 | 0.6374 | 0.8809 | 0.6275 |
| 6-6 | 0.8838 | 0.6368 | 0.8922 | 0.6413 |
| 7-7 | 0.8767 | 0.6121 | 0.8830 | 0.6248 |
| 5-6 | 0.8846 | 0.6458 | 0.8824 | 0.6326 |
| 6-5 | 0.8827 | 0.6328 | 0.8868 | 0.6373 |
| 5-7 | **0.8865** | **0.6476** | **0.9006** | **0.6732** |
| 7-5 | 0.8766 | 0.6135 | 0.8728 | 0.5950 |
| 6-7 | 0.8841 | 0.6398 | 0.8831 | 0.6331 |
| 7-6 | 0.8768 | 0.6133 | 0.8675 | 0.5906 |

Table S3. Results of MGMA with different α values on verification and Test_60 datasets.

| α | Verification dataset | | Test_60 dataset | |  |
| --- | --- | --- | --- | --- | --- |
|  | AUROC | AUPRC | AUROC | AUPRC |  |
| 0.25 | 0.8865 | 0.6476 | 0.9006 | 0.6732 |  |
| 0.5 | 0.8845 | 0.6416 | 0.8980 | 0.6699 | |
| 0.75 | 0.8847 | 0.6425 | 0.8977 | 0.6632 |  |

Table S4. Results of MVMA-PPIS, GHGPR-PPIS and AGAT-PPIS on a Specific Protein (PDB ID: 3q87, Chain A and PDB ID: 2v9t, Chain B)

| **PDB ID** | **Methods** | **TP** | **TN** | **FP** | **FN** |
| --- | --- | --- | --- | --- | --- |
| 3q87, Chain A | AGAT-PPIS | 26 | 75 | 20 | 1 |
|  | GHGPR-PPIS | 27 | 77 | 18 | 0 |
|  | MVMA-PPIS | 27 | 85 | 10 | 0 |
| 2v9t, Chain B | AGAT-PPIS | 18 | 50 | 34 | 5 |
|  | GHGPR-PPIS | 14 | 58 | 26 | 9 |
|  | MVMA-PPIS | 18 | 78 | 6 | 5 |


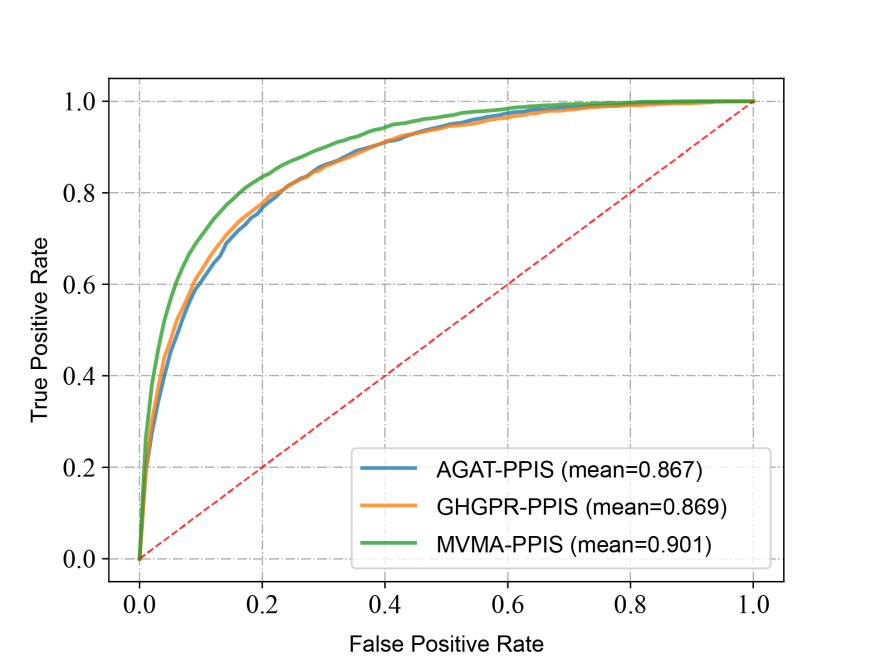


Figure S1 ROC curves of MGMA and other two comparation methods on Test_60 dataset


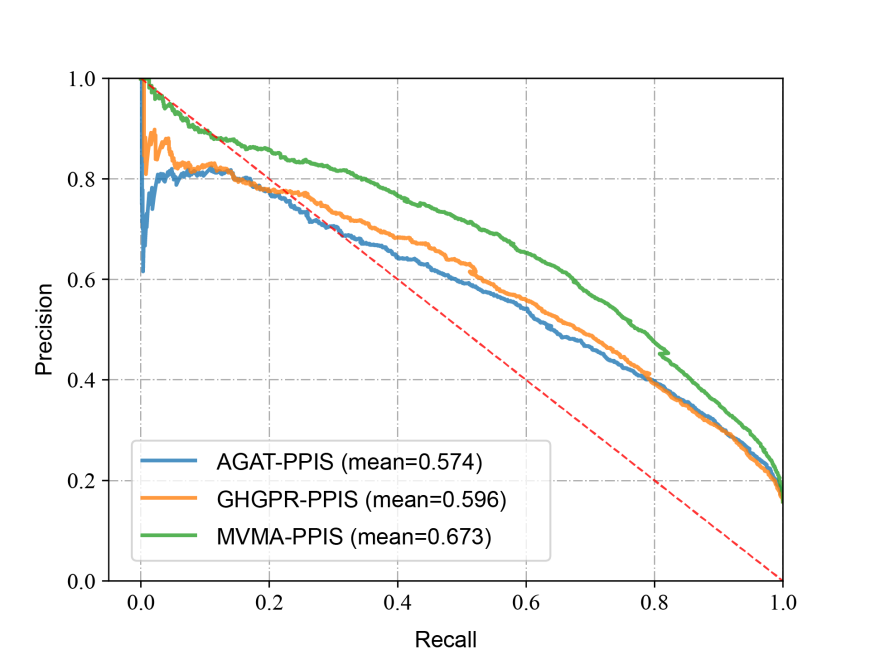


Figure S2 PR curves of MGMA and other two comparation methods on Test_60 dataset


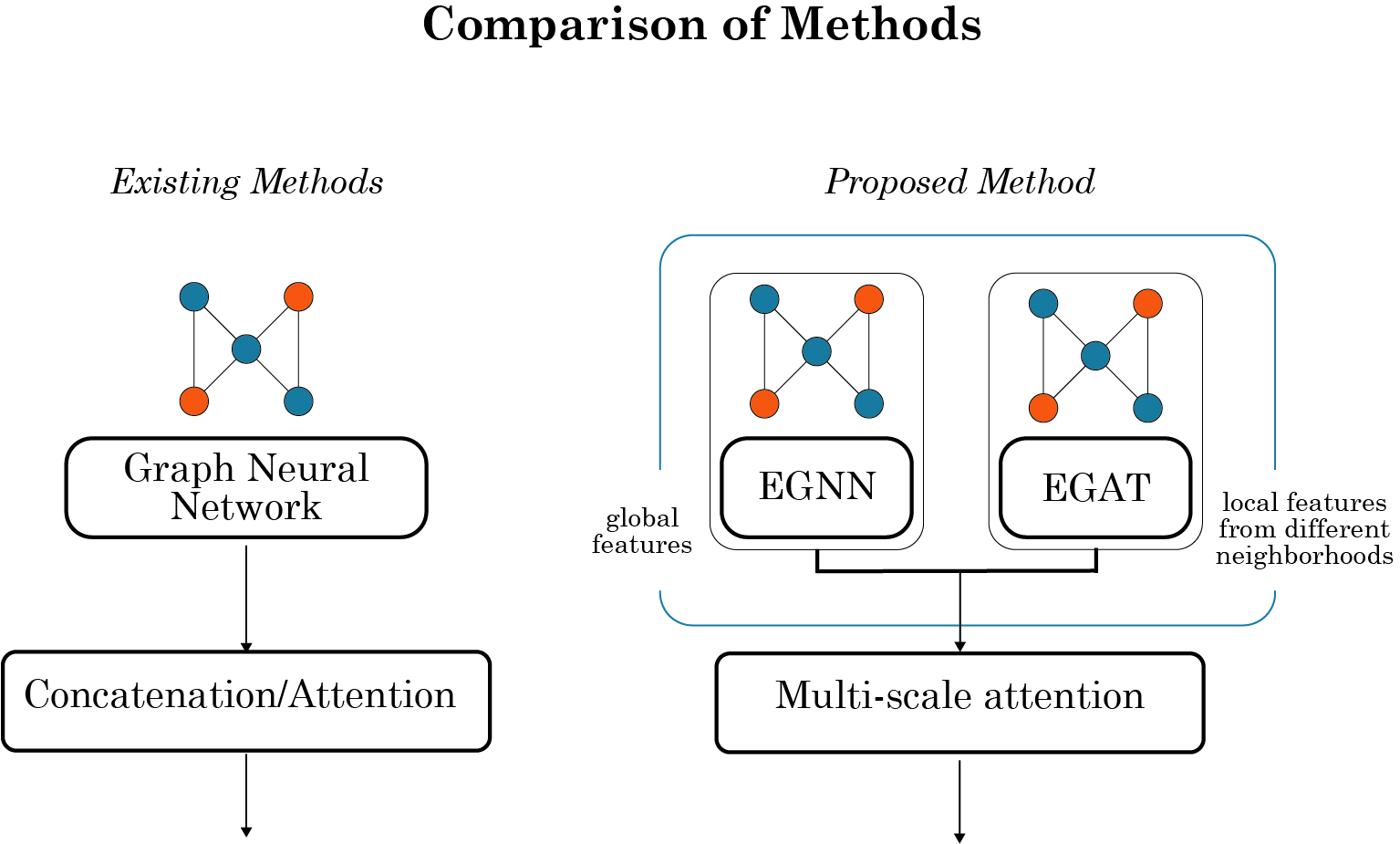


Figure S3. Diagrammatic sketch of framework comparison between our MGMA-PPIS (right) with other existing methods (left). Unlike the single-view GNNs that employ simple feature concatenation or standard self-attention mechanisms, MGMA-PPIS extracts complementary local features across multiple neighborhood scales through EGAT, integrates global structural features via EGNN, and achieves multi-view and multi-scale information fusion using an multi-scale attention mechanism.


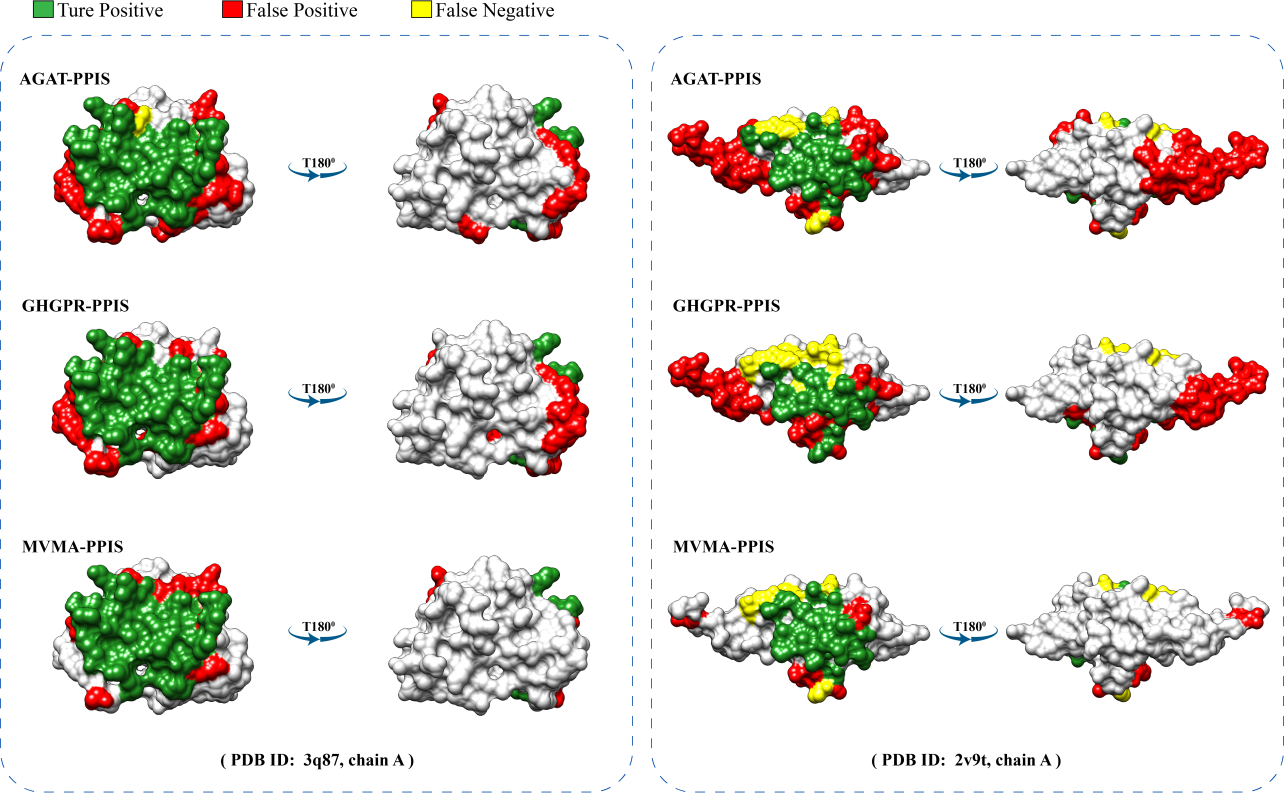


Figure S4. Visualization of prediction results of AGAT-PPIS, GHGPR-PPIS, and MVMA-PPIS on specific protein samples.
