# Supplementary material for: MGMA-PPIS: Predicting the protein–protein interaction site with multiview graph embedding and multiscale attention fusion
Source: Gigascience. 2025 Oct 1;14:giaf114. doi: 10.1093/gigascience/giaf114 (PMC12486388; doi:10.1093/gigascience/giaf114)
Supplement: giaf114_GIGA-D-25-00221_Original_Submission [file giaf114_giga-d-25-00221_original_submission.pdf]

GigaScience

MGMA-PPIS: Predicting protein-protein interaction site with multi-view graph embedding and multi-scale attention fusion

--Manuscript Draft--

**Manuscript Number:**	GIGA-D-25-00221
**Full Title:**	MGMA-PPIS: Predicting protein-protein interaction site with multi-view graph embedding and multi-scale attention fusion
**Article Type:**	Research
**Funding Information:**	National Natural Science Foundation of China (62173271) — Professor Shao-Wu Zhang<br>National Natural Science Foundation of China (62473312) — Professor Shao-Wu Zhang<br>National Natural Science Foundation of China (62172338) — Professor Shao-Wu Zhang
**Abstract:**	Protein-protein interactions (PPIs) play a crucial role in numerous biological processes. Accurate identification of protein-protein interaction sites is critical for a comprehensive understanding of protein functions and pathological mechanisms. However, conventional experimental approaches for detecting PPIS are often time-consuming and labor-intensive, thereby motivating the development of efficient computational methods to identify PPI sites. In this work, we propose a novel graph neural network-based method (called MGMA-PPIS) to predict PPI sites by adopting Multi-view Graph embedding and Multi-scale Attention fusion. MGMA-PPIS integrates global node features extracted by equivariant graph neural network and multi-scale local node features extracted by edge graph attention network across different neighborhood scales, thereby constructing a multi-view graph feature representation. Then, a multi-scale attention network is employed to perform deep feature fusion across multiple scales for achieving high-precision prediction of PPI sites. Experimental results on benchmark datasets show that our MGMA-PPIS outperforms other state-of-the-art methods, and it can effectively predict PPI sites.
**Corresponding Author:**	Shao-Wu Zhang, Ph.D<br>Northwestern Polytechnical University<br>Xian, CHINA
**Corresponding Author Secondary Information:**	
**Corresponding Author's Institution:**	Northwestern Polytechnical University
**Corresponding Author's Secondary Institution:**	
**First Author:**	Yong Han
**First Author Secondary Information:**	
**Order of Authors:**	Yong Han<br>Shao-Wu Zhang, Ph.D<br>Qing-Qing Zhang<br>Ming-Hui Shi
**Order of Authors Secondary Information:**	
**Additional Information:**	
**Question**	**Response**
Are you submitting this manuscript to a	No

Powered by Editorial Manager® and ProduXion Manager® from Aries Systems Corporation

special series or article collection?	
**Experimental design and statistics**<br><br>Full details of the experimental design and statistical methods used should be given in the Methods section, as detailed in our Minimum Standards Reporting Checklist. Information essential to interpreting the data presented should be made available in the figure legends.<br><br>Have you included all the information requested in your manuscript?	Yes
**Resources**<br><br>A description of all resources used, including antibodies, cell lines, animals and software tools, with enough information to allow them to be uniquely identified, should be included in the Methods section. Authors are strongly encouraged to cite Research Resource Identifiers (RRIDs) for antibodies, model organisms and tools, where possible.<br><br>Have you included the information requested as detailed in our Minimum Standards Reporting Checklist?	Yes
**Availability of data and materials**<br><br>All datasets and code on which the conclusions of the paper rely must be either included in your submission or deposited in publicly available repositories (where available and ethically appropriate), referencing such data using a unique identifier in the references and in the “Availability of Data and Materials” section of your manuscript.<br><br>Have you have met the above requirement as detailed in our Minimum Standards Reporting Checklist?	Yes

GigaScience has policies and guidelines in place for the use of generative AI-writing tools such as ChatGPT. If you have used such writing tools to assist with writing the manuscript this must be declared and cited in the text. Authors should not list AI-writing tools and other AI-assisted technologies as an author or co-author and should acknowledge that they are fully responsible for text generated or refined by AI-writing tools.<p><br><br>A summary of use (particularly in the introduction or among methods) needs to be included at the end of the paper, and the outputs should also be included as a supplementary file hosted in GigaDB or other open repositories. Please read our guidelines for more information. <p><br><br>By submitting to GigaScience, you are aware of the journal's AI-writing tools policy, and if you have declared use of such tools below, you have acknowledged this where appropriate in your manuscript and have made a summary of use and outputs available. <p><br><b>AI-assisted writing tools have been used in the preparation of this manuscript?	No

Powered by Editorial Manager® and ProduXion Manager® from Aries Systems Corporation

Manuscript Click here to access/download;Manuscript;MGMA.docx

MGMA-PPIS: Predicting protein-protein interaction site with multi-view graph embedding and multi-scale attention fusion

Yong Han[1,2], Shao-Wu Zhang[1*], Qing-Qing Zhang[1], Ming-Hui Shi[1]

1. MOE Key Laboratory of Information Fusion Technology, School of Automation, Northwestern Polytechnical University, Xi'an, 710072, China

2.Henan Judicial Police Vocational College, Zhengzhou, 450046, China

*Corresponding author. Email: zhangsw@nwpu.edu.cn

Abstract

Protein-protein interactions (PPIs) play a crucial role in numerous biological processes. Accurate identification of protein-protein interaction sites is critical for a comprehensive understanding of protein functions and pathological mechanisms. However, conventional experimental approaches for detecting PPIS are often time-consuming and labor-intensive, thereby motivating the development of efficient computational methods to identify PPI sites. In this work, we propose a novel graph neural network-based method (called MGMA-PPIS) to predict PPI sites by adopting Multi-view Graph embedding and Multi-scale Attention fusion. MGMA-PPIS integrates global node features extracted by equivariant graph neural network and multi-scale local node features extracted by edge graph attention network across different neighborhood scales, thereby constructing a multi-view graph feature representation. Then, a multi-scale attention network is employed to perform deep feature fusion across multiple scales for achieving high-precision prediction of PPI sites. Experimental results on benchmark datasets show that our MGMA-PPIS outperforms other state-of-the-art methods, and it can effectively predict PPI sites.

1. introduction

Proteins perform a wide variety of cellular functions within cells and play key roles in diverse biological processes such as signal transduction, transport, and metabolism [1]. However, proteins rarely function alone; in over 80% of cases, they act as part of complexes [2]. Therefore, studying protein-protein interactions can help building protein interaction networks [3], predicting protein functions [4], providing insights into disease mechanisms [5], and paving the way for drug development and design [6]. Protein-protein interaction (PPI) sites refer to the interface residues of proteins involved in these interactions. Identifying these sites is crucial for unraveling cellular processes and advancing novel drug discovery [7]. However, experimental identification of PPI sites through wet-lab methods, such as coimmunoprecipitation [8] and two-hybrid screening [9], is increasingly impractical due to high time and cost demands. Consequently, it is particularly necessary to develop efficient computational methods as powerful guides and complements to genetic and biochemical experiments.

To date, a variety of computational methods have been developed to predict PPI sites. Early PPI sites prediction approaches primarily rely on machine learning, such as Naive Bayes classifiers [10], LightGBM [11], Random Forests[12, 13], and XGBoost [14, 15]. These methods employ the

feature engineering to select appropriate features, such as raw protein sequences, position-specific scoring matrices (PSSM) and definitions of secondary protein structures (DSSP), to represent proteins, and then use machine learning algorithms to predict PPI sites. Recent years, researchers have increasingly turned their attention to deep learning algorithms, which have further improved prediction accuracy of PPI sites [16, 17]. Existing deep learning methods for predicting PPI sites can be broadly categorized into sequence-based methods (e.g., convolutional neural networks and recurrent neural networks), and structure-based methods (e.g., graph neural networks). Convolutional neural network (CNN)-based methods, such as DeepPPISP [17] and ProB-site [18], capture the local contextual features of protein sequences through convolution operations to predict PPI sites. DeepPPISP [17] first extracts the local contextual features from the neighboring amino acids of a target residue by using a sliding window approach, and extracts the global features from protein sequences using TextCNN. Then, the local contextual features and the global features are integrated together and fed them into a fully connected (FC) layer for predicting PPI sites. ProB-site [18] utilizes sub-CNN architecture to extract three separate higher-order features from sequential information of proteins, and feeds them into the FC layer to achieve accurate prediction of PPI sites. Although these CNN-based methods perform well, they often overlook the hidden long-range dependencies within protein sequences. In contrast, RNN-based methods, such as DELPHI [19] and DLPred [20], are capable of handling long-range dependencies and global information from protein sequences, enabling more robust representation of long-distance correlations in protein sequences. DELPHI [19] uses the high-scoring segment pairs (HSPs), position information, and 3-mer amino acid embedding as three novel features, then build an ensemble framework with the CNN and RNN for predicting PPI sites. DLPred [20] employ a deep learning architecture based on Simplified Long Short-Term Memory (SLSTM) networks to enhance the prediction performance of PPI sites. With the emergence of GNNs, structure-based PPI site prediction methods that utilize protein tertiary structural features have advanced, enabling the accurate extraction of protein features from protein structures. In particular, the growing availability of protein tertiary structure data and the advent of high-accuracy structure prediction tools like AlphaFold2 [21] have significantly advanced the application of GNNs in PPI sites prediction, yielding some PPI sites prediction methods, such as AGAT-PPIS [16], GraphPPIS [22], AGF-PPIS [23] and GHGPR-PPIS [24]. AGAT-PPIS [16] integrates edge features to calculate attention scores for refining node embeddings, thereby facilitating the prediction of PPI sites. GraphPPIS [22] adopts a deep graph convolutional neural network (GCN) framework to predict PPI sites by incorporating initial residual connections and identity mapping techniques. AGF-PPIS [23] leverages the multi-head self-attention mechanisms, graph convolutional networks, and feedforward neural networks to extract protein features, which are subsequently inputted into a multi-layer perceptron (MLP) for achieving PPI sites prediction. GHGPR-PPIS [24] first constructs a graph network by using a heat kernel-based graph convolutional network, then combines the generalized PageRank approach with an edge self-attention feature processing trick to extract features that are inputted into a MLP for PPI sites

prediction. Although above GNN-based methods have achieved good performance, they often rely on a single encoder, which hinders their ability to comprehensively extract information from complex proteins. Meanwhile, with the rise of large language models such as the classic Transformer [25], attention mechanisms have garnered increasing attention, which are used to enhance the performance of GCNs [26]. However, they employ a traditional self-attention mechanism, which often encounters problems such as excessive concentration or dispersion of attention [27], resulting in inadequate information representation, thereby hindering the model's ability to accurately interpret the original input.

In this work, we proposed a novel computational method (named MGMA-PPIS) to predict PPI sites by introducing Multi-view Graph embeddings and Multi-scale Attention-based feature fusion mechanism. MGMA-PPIS aims to enhance existing graph neural network-based methods for high-precision prediction of PPI sites. In MGMA-PPIS, each protein sequence is represented as a graph, where amino acids are treated as nodes, thereby transforming the PPI site prediction task into a graph node classification problem. Specifically, MGMA-PPIS first construct an adjacency matrix for the protein graph by calculating the Euclidean distances between amino acids, and integrate both sequence and structure information to generate representations for nodes and edges. Then, MGMA-PPIS employs an Edge Graph Attention Network (EGAT) and an E(n) Equivariant Graph Neural Network (EGNN) to extract multi-ranges local and global embeddings of amino acid nodes, and the multi-scale attention mechanism is subsequently applied at the amino acid level to fuse feature embeddings, enabling more effective capture of key information. Moreover, MGMA-PPIS utilize the focal loss function [23, 28] to optimize the model and mitigate the impact of class imbalance. To the best of our knowledge, this is the first attempt to apply multi-view graph embedding fusion to PPI sites prediction. Comprehensive evaluations on multiple benchmark datasets and independent test sets demonstrate that MGMA-PPIS significantly outperforms other existing methods.

2. Materials and methods

2.1. Datasets

In this work, we utilize the same benchmark dataset as previous work of AGAT-PPIS[16] for parameter tuning and model's performance testing. AGAT-PPIS dataset is derived from the GraphPPIS dataset, which includes the following subsets: one training set of Train_335-1, three test sets of Test_315-28, Test_60-0, and Ubtest_31-6. The statistical information of the AGAT-PPIS datasets is shown in Supplementary Table S1.

2.2. Protein representation

In MGMA-PPIS-PPIS framework, we use an undirected graph $\mathbf{G}=(\mathbf{V},\mathbf{A},\mathbf{E})$ to represent each protein. Here, $\mathbf{V}=\{v_i\}$ is amino acid residue node set, $v_i \in \mathbb{R}^{\mathrm{D}_v}$ is the feature vector of node i $(i \in \mathrm{N}_v)$, N_v is the number of amino acid residues contained in a protein; $\mathbf{A} \in \mathbb{R}^{\mathrm{N}_v \times \mathrm{N}_v}$ is the

adjacency matrix of the graph **G**; $\mathbf{E} \in \{\mathbf{e}_{ij} \mid \mathbf{A}_{ij} = 1\}$ is edge set, and $\mathbf{e}_{ij}$ is the feature vector of edge between nodes i and j. That is, the elements of **E** are determined by the adjacency matrix **A**: if $\mathbf{A}_{ij}$ = 1, then $\mathbf{e}_{ij} \in \mathbf{E}$; if $\mathbf{A}_{ij}$ = 0, then $\mathbf{e}_{ij} \notin \mathbf{E}$.

2.2.1 Node representations

In protein graphs, the amino acid node features are derived from protein sequence and structure, and their extraction process follows the AGAT-PPIS[16]. All amino acid node feature vectors are combined together to form an amino acid node feature matrix $\mathbf{X}_{node}$. Table 1 describe the features of these animo acids.

Table1. Summary of animo acid node features

Features	Dimension	Category	Description
PSSM	20	Sequence information	Position-Specific Scoring Matrix
HMM	20	Sequence information	Hidden Markov Models matrix
DSSP	14	Structure information	Define Secondary Structure of Proteins
AF	7	Structure information	Atomic Features
PPE	1	Structure information	Pseudo-Position Embedding

Protein sequence features

The Position-Specific Scoring Matrix (PSSM) and the Hidden Markov Model Matrix (HMM) are important features used to characterize the evolutionary information of protein sequences. The PSSM is generated using the PSI-BLAST v2.10.1 tool [29], while the HMM matrix is constructed using the HHblits v3.0.3 algorithm [30]. To further optimize the feature representation, the original values in the matrices are normalized to standardized scores ranging between 0 and 1, ultimately resulting in the feature matrices $\mathbf{X}_{\mathrm{PSSM}} \in \mathbb{R}^{\mathrm{N}_v \times 20}$ and $\mathbf{X}_{\mathrm{HMM}} \in \mathbb{R}^{\mathrm{N}_v \times 20}$, respectively.

Protein structure features

Protein structural features include protein secondary structure (DSSP) features, atomic features (AF), and pseudo-position features (PPE). DSSP features are generated using the DSSP algorithm [31], which are composed of 14-dimensional residue features. Specifically, the first 9 features are represented in one-hot encoding format to indicate the secondary structure states of the protein chain, covering the following 9 secondary structure types: H (α-helix), G (310-helix), I (π-helix), E (extended strand), B (isolated bridge), T (turn), S (bend), and C (other or unknown secondary structures). The next 4 features are derived by applying sine and cosine transformations to the backbone torsion angles PHI and PSI of the peptide chain. Another one feature is generated by converting the solvent accessible surface area into relative solvent accessibility. Ultimately, 14 features are combined together to form the structural feature matrix $\mathbf{X}_{\mathrm{DSSP}} \in \mathbb{R}^{\mathrm{N}_v \times 14}$ for representing one protein, here N_v is the number of amino acid residues contained in a protein.

Atomic Features (AF) are the seven attributes of each non-hydrogen atom in a residue,

including: atomic mass, B factor, whether it is a residue side chain atom, electron charge, the number of hydrogen atoms bonded to it, whether it is part of a ring, and the van der Waals radius of the atom. Since the number of atoms in each residue may vary, to standardize the feature representation, the average values of the seven features for all atoms in each residue are calculated. Therefore, 7 features are combined together to form the atomic feature matrix $\mathbf{X}_{\mathrm{AF}} \in \mathbb{R}^{\mathrm{N}_v \times 7}$ for representing one protein.

Pseudo-Position Embedding (PPE) feature of amino acid residues is used to characterize the relative positional information of each residue with respect to a reference residue. In this work, the coordinates of the side chain centroid (SC) are adopted as the pseudo-position representation of residues, thereby generating the pseudo-position embedding matrix $\mathbf{X}_{\mathrm{PPE}} \in \mathbb{R}^{\mathrm{N}_v \times 1}$.

The protein sequence feature matrices (i.e., $\mathbf{X}_{\mathrm{PSSM}}$, $\mathbf{X}_{\mathrm{HMM}}$) are concatenated with the structure feature matrices (i.e., $\mathbf{X}_{\mathrm{DSSP}}$, $\mathbf{X}_{\mathrm{AF}}$, $\mathbf{X}_{\mathrm{PPE}}$) to form a unified feature matrix $\mathbf{X}_{node} \in \mathbb{R}^{\mathrm{N}_v \times 62}$ for represent one protein.

$$\mathbf{X}_{node} = \left[\mathbf{X}_{\mathrm{PSSM}}, \mathbf{X}_{\mathrm{HMM}}, \mathbf{X}_{\mathrm{DSSP}}, \mathbf{X}_{\mathrm{AF}}, \mathbf{X}_{\mathrm{PPE}}\right]$$

2.2.2 Edge representations

Edge features in protein graphs focus on the spatial relationships between amino acid nodes. We extract the positional data of amino acids from PDB file, and calculate the Euclidean distance between them. By setting a distance threshold, we evaluate whether two amino acid nodes meet specific relationship criteria. If the distance is below the threshold, we create an edge between these two amino acid nodes. According to the reference [16], we set the cutoff distance hyperparameter to 14 Å, thus we can obtain an adjacency matrix **A** of a protein, where $\mathbf{A}_{ij} = 1$ indicates the presence of an edge between node *i* and node *j*, and $\mathbf{A}_{ij} = 0$ indicates no edge.

The computation of edge features involves two types of positional encoding, that is Euclidean distance between two nodes and the cosine value of the angle between two nodes, thus we can obtain the edge feature matrix $\mathbf{X}_{edge} \in \mathbb{R}^{\mathrm{N}_e \times 2}$, here N_e represents the number of edges in protein graph.

2.3 MGMA-PPIS

In this work, we formulate the prediction of protein-protein interaction (PPI) binding sites as a graph node classification task, and innovatively propose a feature fusion framework (named MGMA-PPIS) that combines multi-view graph embeddings with a multi-scale attention mechanism to predict PPI sites. The overall architecture of MGMA-PPIS is illustrated in Figure 1. MGMA-PPIS consists of three main components: the input module, the feature extraction module, and the output module (Figure 1a). First, MGMA-PPIS constructs protein graph, and extract the node features and edge

features from protein sequence and structure, respectively. Subsequently, protein graph with node and edge features is fed into the feature extraction module that consists of an E(n) Equivariant Graph Neural Network (EGNN)[32] with residual connections and an Edge Graph Attention Network (EGAT)[33] with residual connections. By concatenating the global node features extracted from EGNN with the multi-scale local node features extracted from EGAT at different neighborhood scales through a MUlti-Scale attEntion network (MUSE), we can obtain the node embeddings of amino acids in protein graph. In the end, these node embeddings are fed into a Multilayer Perceptron (MLP) to output the prediction results of PPI sites.

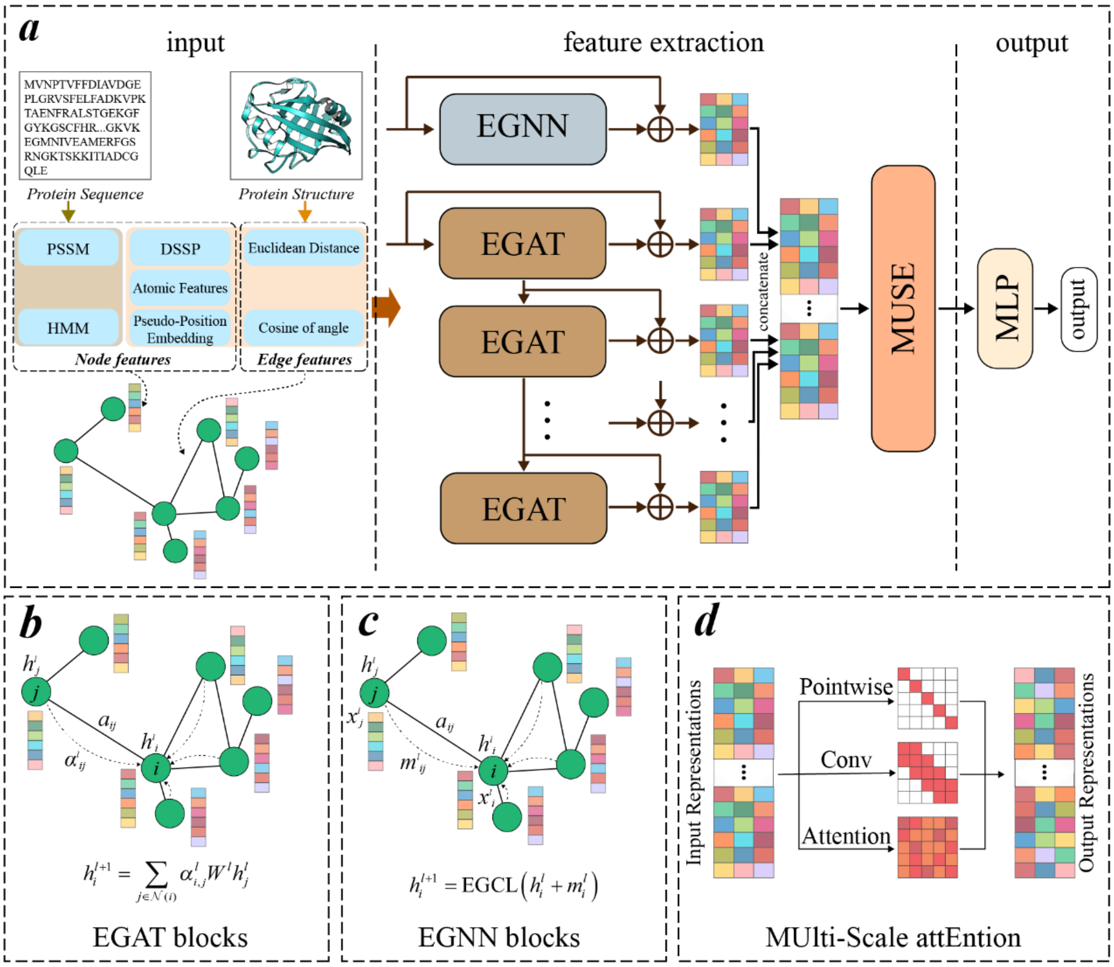

Figure 1 Overview of MGMA-PPIS framework. a. MGMA-PPIS consists of three principal components: input module, feature extraction module, and output module. In input module, protein sequence and its structure are utilized to derive node features, edge features, and an adjacency matrix for building a protein graph with node/edge representation. In feature extraction module, the protein graphs are processed using EGNN and EGAT to extract multi-view graph features, which are integrated via the MUSE. In output module, MLP is employed to further refine the node embeddings to output the prediction results of PPI sites. b. EGAT module, in which protein graph is processed through EGAT with residual connections to extract multi-scale local node features across various neighborhood levels. c. EGNN module, in which protein graph is processed through EGNN with residual connections to capture the global node features. d. MUlti-Scale attEntion Network, which

integrates self-attention mechanisms, depthwise separable convolutions, and position-wise feedforward networks to enhance the feature representation.

2.3.1. Edge Graph attention network

Graph Attention Network (GAT)[34] extends Graph Neural Networks (GNNs)[35] by not only defining local interactions through edges but also leveraging them to store temporary attention scores (i.e., edge weights) that quantify and adjust the importance of each connection. The initial idea of GAT is to compute independent attention scores for each edge in the graph, enabling GAT to learn which connections are crucial for information propagation between nodes and their neighbors. However, in GAT implementation, the intrinsic information of edges is neither adequately considered nor learned, and cannot be effectively propagated through the network, thereby limiting the capacity of GAT for representation. To address this limitation, the Edge Graph Attention Network (EGAT) extends GAT by effectively incorporating edge features, enhancing its capability to model graph-structured data. EGAT updates the edge features $\mathbf{e}_{ij}^{l+1}$ in l+1 layer using the node features $\mathbf{h}_i^l$ and edge features $\mathbf{e}_{ij}^l$ at layer l, then utilizes $\mathbf{e}_{ij}^{l+1}$ to compute edge weights of the neighboring nodes, aggregating their node features to update the node features $\mathbf{h}_i^{l+1}$. Consequently, EGAT can more efficiently learn the local information of protein from neighboring nodes based on the attention weights. As the number of layers increases, EGAT is capable of capturing the features from higher-order neighboring nodes, thus obtaining local information over a broader range. However, as the depth of a graph neural network (GNN) increases, the over-smoothing phenomenon often occurs, causing the features of different nodes in the graph to become indistinguishable. To mitigate this issue, residual connections are introduced between EGAT layers to enhance the extraction of deep node features, thereby enabling more effective capture of key structural information of protein graph. The update rules for node features and edge features are defined as follows:

$$\mathbf{e}_{ij}^{l+1} = LeakyReLU(\mathbf{A}^l\left[\mathbf{h}_i^l \,||\, \mathbf{e}_{ij}^l \,||\, \mathbf{h}_j^l\right]),\ \ l=1,2,\cdots,Z$$

$$\alpha_{ij}^l = \text{Softmax}(\vec{F}^l(\mathbf{e}_{ij}^{l+1}))$$

$$\mathbf{h}_i^{l+1} = ReLU(\mathbf{h}_i^l + \sum_{j\in\mathcal{N}(i)} \alpha_{ij}^l \mathbf{W}^l \mathbf{h}_j^l)$$

where, symbol ($\bullet||\bullet$) denotes the vector concatenation operation; $\mathbf{e}_{ij}^{l+1}$ is the updated edge feature; $\mathbf{h}_i^l$ is the features of node i in l layer; $\mathbf{h}_j^l$ is the features of node j in l layer; $\mathbf{e}_{ij}^l$ is the edge feature between nodes i and j in l layer; **A** is a learnable matrix**;** *LeakyReLU* is a activation function. The attention weight coefficient α_{ij} between node i and node j is derived from the updated edge feature $\mathbf{e}_{ij}^{l+1}$ using a learnable weight vector $\vec{F}$ and normalized via the Softmax function. The updated node feature $\mathbf{h}_i^{l+1}$ is obtained by performing a weighted summation of the neighboring

node features $\mathbf{h}^l_j$ based on the attention weight coefficients α_{ij}, and adding the result before updating $\mathbf{h}^l_i$, followed by a *ReLU* activation function.

In this work, we process the node feature matrix $\mathbf{X}_{node}$ through a fully connected (FC) network to obtain the node embedding feature matrix $\mathbf{X}^{\mathrm{EGAT}}_{node} = [\mathbf{h}^0_1, \mathbf{h}^0_2, \cdots, \mathbf{h}^0_{\mathrm{N}_v}]^{\mathrm{T}}$, $\mathbf{X}^{\mathrm{EGAT}}_{node} \in \mathbb{R}^{\mathrm{N}_v \times 128}$ and the node embedding feature matrix $\mathbf{X}^{\mathrm{EGAT}}_{node}$ and the edge matrix $\mathbf{X}_{edge}$ are used as the inputs of EGAT. Then, we treat the output $\mathbf{h}^l_i$ of EGAT at different layers as the local features of nodes, and concatenate these features together to form the multi-scale local feature $\mathbf{h}^{local}_i$ of node *i*.

$$\mathbf{h}^{local}_i = [\mathbf{h}^1_i \,||\, \mathbf{h}^2_i \,||\cdots||\, \mathbf{h}^L_i]$$

where *L* is the total number of layers in EGAT, and symbol (•||•) denotes the vector concatenation operation

2.3.2. Equivariant graph neural network

The Equivariant Graph Neural Network (EGNN) introduces a novel architecture that is equivariant to translation, rotation, reflection (E(n)), and permutation, serving as a variant of GNN. Therefore, leveraging EGNN for protein feature extraction can better capture its structural properties. The network architecture is composed of a stack of Equivariant Graph Convolutional Layers (EGCLs). EGCL updates the node coordinate features $\mathbf{x}^{l+1}_i$ and node features $\mathbf{h}^{l+1}_i$ for the next layer by aggregating edge features $\mathbf{e}_{ij}$ (between node *i* and node *j*) and combining the node coordinate features $\mathbf{x}^l_i$ and node features $\mathbf{h}^l_i$ from the current layer. The update rules for node coordinates and features are defined as follows:

$$\mathbf{m}^l_{ij} = \phi_e\left(\mathbf{h}^l_i, \mathbf{h}^l_j, \left\|\mathbf{x}^l_i - \mathbf{x}^l_j\right\|^2, \mathbf{e}_{ij}\right)$$

$$\mathbf{x}^{l+1}_i = \mathbf{x}^l_i + C\sum_{j\neq i}(\mathbf{x}^l_i + \mathbf{x}^l_j)\phi_x(\mathbf{m}^l_{ij})$$

$$\mathbf{m}^l_i = \sum_{j\neq i}(\mathbf{m}^l_{ij})$$

$$\mathbf{h}^{l+1}_i = ReLU(\mathbf{h}^l_i + \phi_h(\mathbf{h}^l_i + \mathbf{m}^l_i))$$

where, $\mathbf{m}^l_{ij}$ represents the edge embedding between node *i* and node *j* at layer *l*; ϕ_e is an edge operation; ϕ_x is a coordinate operation; $C = 1/(M-1)$ is a constant factor, and *M* is the total number of nodes in the graph; ϕ_h is a node operation.

When updating coordinates and node features, EGNN integrates the node coordinates, node information, and edge information of the entire graph to capture more global information. In contrast

to EGAT that primarily captures the local information, EGNN is more adept at capturing the global information. Therefore, we use EGNN to extract the global features of proteins.

Similarly, we process the node feature matrix $\mathbf{X}_{node}$ through a FC network to generate the node embedding feature matrix $\mathbf{X}_{node}^{\mathrm{EGNN}} = [\mathbf{h}_1^0, \mathbf{h}_2^0, \cdots, \mathbf{h}_{\mathrm{N}_v}^0]^{\mathrm{T}}$, $\mathbf{X}_{node}^{\mathrm{EGNN}} \in \mathbb{R}^{\mathrm{N}_v \times 256}$, then input the node embedding feature matrix $\mathbf{X}_{node}^{\mathrm{EGNN}}$ and edge matrix $\mathbf{X}_{edge}$ into EGNN. The output $\mathbf{h}_i^{global} = \mathbf{h}_i^{\mathrm{Y}}$ of last layer is taken as the global features of nodes, where Y is the number of layers in EGNN. Finally, we concatenate the global feature $\mathbf{h}_i^{global}$ extracted by EGNN with the multi-scale local features $\mathbf{h}_i^{local}$ extracted by EGAT to generate the final node feature vector $\mathbf{h}_i^{all}$ of node *i*, $\mathbf{h}_i^{all} = [\mathbf{h}_i^{global} \,||\, \mathbf{h}_i^{local}]$. Thereby, we obtain the multi-view feature embedding matrix $\mathbf{X}_{embedding}$ of one protein, $\mathbf{X}_{embedding} = [\mathbf{h}_1^{all}, \mathbf{h}_2^{all}, \cdots, \mathbf{h}_{\mathrm{N}_v}^{all}]^{\mathrm{T}}$.

2.3.3. Multi-scale attention network

To focus on important features, we introduce the attention-based feature representation learning to process protein sequences. In sequence learning, the self-attention mechanism has proven to be highly efficient and has achieved significant performance improvements across numerous tasks[xx]. However, the attention in deep layers often overly focuses on a single token, which not only limits the full utilization of local information, but also shows inadequacies when representing long sequences. Therefore, to more effectively capture both long-range and short-range linguistic structures, the multi-scale attention mechanism with parallelization is proposed for parallel multi-scale representation learning on sequential data [36]. Therefore, we integrate convolution operations and self-attention mechanisms to encode protein sequences in parallel across multiple scales, embodying the core principle of parallel multi-scale sequence representation learning. Our multi-scale attention network module consists of three main components: self-attention for capturing the global contextual features, depth-wise separable convolution for extracting the local patterns, and position-wise feed-forward network for capturing the token features.

By leveraging the characteristics of MUSE in sequence representation learning, we adopt MUSE to the multi-view feature embedding matrix $\mathbf{X}_{embedding}$, and use a fusion trick to output a representation $\mathbf{X}_{muse}$.

$$\mathbf{X}_{muse} = \mathbf{X}_{embedding} + \mathrm{Attention}(\mathbf{X}_{embedding}) + \mathrm{Conv}(\mathbf{X}_{embedding}) + \mathrm{Pointwise}(\mathbf{X}_{embedding})$$

where, “Attention” is the self-attention operation; “Conv” is the depth-wise separable convolution operation; “Pointwise” is the position-wise feed-forward operation.

The self-attention responsible for learning global contextual representations projects the multi-scale feature matrix $\mathbf{X}_{embedding}$ into three distinct representations: key *K*, query *Q*, and

value V, for computing the output representation.

$$\text{Attention}(\mathbf{X}) = \sigma(\mathbf{Q},\mathbf{K},\mathbf{V})W^O$$

$$\sigma(\mathbf{Q},\mathbf{K},\mathbf{V}) = \text{softmax}\left(\mathbf{Q}\mathbf{K}^{\mathrm{T}} / \sqrt{d_k}\right)\mathbf{V}$$

$$\mathbf{Q} = \mathbf{X}_{embedding}W^Q,\ \mathbf{K} = \mathbf{X}_{embedding}W^K,\ \mathbf{V} = \mathbf{X}_{embedding}W^V$$

where, W^Q, W^K, W^V and W^O are the projection parameters; $\sigma(\bullet)$ is the self-attention operation, which is defined as the dot product between the key, query, and value pairs.

The depth-wise separable convolution as the convolutional component (including depth-wise convolution and point-wise convolution) within a parallel structure, can effectively compensate for the insufficient utilization of local information, while the self-attention mechanism focuses on capturing global dependencies[37]. Each convolutional submodule contains multiple units with different kernel sizes to capture features at various scales. The introduced in the work[38]. For an input sequence **X**, the computation of output **O** is formulated as follows:

$$\mathbf{O}_{i,c} = \text{DepthConv}_k(\mathbf{X}) = \sum_{j=1}^{k} W_{c,j} \cdot \mathbf{X}_{(i+j-\lceil (k+1)/2 \rceil),c}$$

where, 'DepthConv' is the depth-wise separable convolution operation; k is the kernel width.

The formulation of point-wise separable convolution with a kernel size of k is as follows:

$$\text{Conv}_k(\mathbf{X}) = \text{DepthConv}_k(\mathbf{V}_2)W^{out}$$

$$\mathbf{V}_2 = \mathbf{X}_{embedding}W^V$$

where, W^V and W^{out} are the projection parameters; W^V is a point-wise projecting matrix.

By adopting shared projections, the input features can be mapped into the same hidden space, enabling better learning of contextual sequence representations[27]. Therefore, the projection operations in both the self-attention mechanism $\mathbf{V} = \mathbf{X}_{embedding}W^V$ and the convolution mechanism $\mathbf{V}_2 = \mathbf{X}_{embedding}W^V$ are shared.

In addition, dynamic convolution (the optimal variant of DepthConv) is employed in this work. Due to automatically assigning the convolution kernels, we apply the following formula to normalize the weights.

$$\text{Conv}(\mathbf{X}) = \sum_{i=1}^{n} \frac{\exp(\alpha_i)}{\sum_{j=1}^{n}\exp(\alpha_j)} \text{Conv}_{k_i}(\mathbf{X})$$

where, α_i denotes the weight of DepthConv output network.

The position-wise feed-forward network is used to capture token representations. To learn token-level feature representations, the parallel structure incorporates a position-wise feed-forward network. Since the linear transformation is identical across different positions, it can be regarded

as a token feature extractor for capturing the feature representation of each token.

$$\text{Pointwise}(\mathbf{X}) = ReLu(\mathbf{X}W_1 + b_1)W_2 + b_2$$

where, W_1, b_1 W_2 and b_2 are the projection parameters of the feed forward neural network.

2.4. Focal loss

Cross-entropy loss (CE) is a traditional loss function that is widely used for binary classification tasks. However, the datasets used in this work exhibit a highly imbalanced class distribution (as shown in Table S1). Such imbalance typically leads to a strong bias, causing the model toward the majority class. When training a model using the cross-entropy loss function, the optimization objective is to minimize the average loss over the entire training dataset. As a result, model tends to perform well on majority class samples but poorly on minority class samples. Therefore, in this work, we adopt focal loss (FL) as the loss function to mitigate the adverse effects of class imbalance on model performance. FL not only assigns asymmetric weights to samples from different classes, but also differentiates between easy and hard examples by applying different weights, which helps reduce the bias introduced during training. Focal loss is derived from the cross-entropy loss (CE):

$$CE(p, y) = \begin{cases} -\log(p) & if\ y = 1 \\ -\log(1-p) & otherwise \end{cases}$$

where, $y \in \{\pm 1\}$ denotes the ground truth for negative and positive classes, respectively; $p \in [0,1]$ represents the estimated probability for the class with label $y = 1$.

By incorporating a modulating factor $(1-p_t)^{\gamma}$ into the CE with an adjustable focusing parameter $\gamma \geq 0$, we reduce the weighting of easy examples to mitigate the influence of simple examples, thereby shifting the training focus toward challenging negatives. Thus, focal loss can be formulated as:

$$\text{FL}(p_t) = -(1-p_t)^{\gamma}\,\text{CE}(p_t) = -\alpha(1-p_t)^{\gamma}\log(p_t)$$

$$p_t = \begin{cases} p & if\ y = 1 \\ 1-p & otherwise \end{cases}$$

where α is a weighting factor, which is used to address the imbalance between positive and negative samples. Focal loss enables the classifier to primarily focus on minority class samples and hard-to-classify examples.

2.5. Evaluation metrics

In this work, we use Accuracy (ACC), Precision, Recall, F1-score (F1), Matthews Correlation Coefficient (MCC), Area Under the Receiver Operating Characteristic Curve (AUROC), and Area Under the Precision-Recall Curve (AUPRC) to evaluate the model performance. The formulas of ACC, Precision, Recall, F1 and MCC are as follows:

$$\text{ACC} = \frac{TP + TN}{TP + TN + FP + FN}$$

$$\text{Precision} = \frac{TP}{TP + FP}$$

$$\text{Recall} = \frac{TP}{TP + FN}$$

$$\text{F1} = \frac{2 \times \text{Precision} \times \text{Recall}}{\text{Precision} + \text{Recall}}$$

$$\text{MCC} = \frac{TP \times TN - FP \times FN}{\sqrt{(TP + FP)(TP + FN)(TN + FP)(TN + FN)}}$$

where, TP and TN represent the number of correctly predicted interaction sites and non-interaction sites, respectively; FP and FN represent the number of incorrectly predicted interaction sites and non-interaction sites, respectively. AUROC and AUPRC are threshold-independent metrics that reflect model overall performance. Given the significant class imbalance between positive and negative samples in our work, we place particular emphasis on three evaluation metrics that are especially important for imbalanced datasets: F1, MCC, and AUPRC, in order to more accurately assess the model performance.

3. Results and discussion

In this work, MGMA-PPIS is implemented using Python 3.9 with PyTorch 2.3.0 and the Deep Graph Library (DGL) 2.4.0 packages. The primary criterion for performance evaluation is the average AUPRC obtained through 5-fold cross-validation test, which is used to guide the selection and optimization of relevant features and hyperparameters. Through experimental validation, we determine the final hyperparameter configurations as follows: five layers EGAT, seven layers EGNN, and α = 0.25. Additionally, according to the insights from previous studies[23] and empirical validation, we set γ =2. The detailed information is presented in Supplementary Tables S2 and S3.

3.1. Performance comparison of MGMA-PPIS with other methods

To comprehensively evaluate the performance of MGMA-PPIS, we first compare our MGMA-PPIS with other existing methods on an independent test dataset (i.e., Test_60). As shown in Table 2, MGMA-PPIS-PPIS outperforms all other methods across the seven metrics. Specifically, compared with the graph convolution-based methods (i.e., AGAT-PPIS, AGF-PPIS and GHGPR-PPIS) with the best prediction results among all comparison methods, our MGMA-PPIS-PPIS increases ACC by 0.023~0.027, Precision by 0.072~0.084, Recall by 0.032~0.049, F1 by 0.053~0.088, MCC by

0.066~0.083, AUROC by 0.031~0.034, AUPRC by 0.074~0.099. These results demonstrate the effectiveness and superiority of MGMA-PPIS in predicting PPI sites. In addition, the AUROC and AUPRC curves of MGMA-PPIS-PPIS, AGAT-PPIS and GHGRP-PPIS are shown in Figures S1 and S2.

Table 2. Result of MGMA-PPIS and other thirteen comparative methods on Test_60 dataset

Method	ACC	Precision	Recall	F1	MCC	AUROC	AUPRC
PSIVER	0.561	0.188	0.534	0.278	0.074	0.573	0.190
ProNA2020	0.738	0.275	0.402	0.326	0.176	N/A	N/A
SCRIBER	0.667	0.253	0.568	0.350	0.193	0.665	0.278
DLPred	0.682	0.264	0.565	0.360	0.208	0.677	0.294
DELPHI	0.697	0.276	0.568	0.372	0.225	0.699	0.319
DeepPPISP	0.657	0.243	0.539	0.335	0.167	0.653	0.276
SPPIDER	0.752	0.331	0.557	0.415	0.285	0.755	0.373
MaSIF-site	0.780	0.370	0.561	0.446	0.326	0.775	0.439
GraphPPIS	0.776	0.368	0.584	0.451	0.333	0.786	0.429
DeepProSite	0.842	0.501	0.443	0.470	0.379	0.813	0.490
AGAT-PPIS	0.856	0.539	0.603	0.569	0.484	0.867	0.574
AGF-PPIS	0.860	0.551	0.620	0.584	0.501	0.870	0.599
GHGPR-PPIS	0.860	0.551	0.620	0.583	0.501	0.869	0.596
MGMA-PPIS	**0.883**	**0.623**	**0.652**	**0.637**	**0.567**	**0.901**	**0.673**

Considering that the performance of AGAT-PPIS, AGF-PPIS and GHGPR-PPIS on Test_60 dataset is significantly better than other nine comparation methods, we compare our MGMA-PPIS with these three methods on other three independent test sets (i.e.,Test_315-28, Btest_31-6, and UBtest_31-6) to further validate the performance of MGMA-PPIS. As shown in Table 3, we can see that the MCC and AUPRC metrics of our MGMA-PPIS are still higher than that of other three methods, indicating that our MGMA-PPIS-PPIS has the superior generalization ability.

Table 3. Results of MGMA-PPIS and other three comparation methods on Test_315-28, BTest_31-6 and UBtest_31-6 datasets.

Method	Test_315-28		Btest_31-6		UBtest_31-6	
	MCC	AUPRC	MCC	AUPRC	MCC	AUPRC
AGAT-PPIS	0.481	0.572	0.485	0.583	0.327	0.365
AGF-PPIS	0.484	0.565	0.518	0.604	0.339	0.370
GHGPR-PPIS	0.486	0.566	N/A	N/A	0.356	0.367
MGMA-PPIS	**0.535**	**0.623**	**0.540**	**0.604**	**0.374**	**0.404**

∗ N/A indicates that the corresponding value is not available in the original literature.

3.2. Ablation experiments of diverse architecture components in MGMA-PPIS

In MGMA framework, EGAT is responsible for extracting local features from different neighborhoods within the protein graph, EGNN captures the global features of the protein graph, and the multi-scale attention network serves as the feature fusion mechanism. To investigate the contributions of these key components for improving MGMA-PPIS performance, we design three variants (i.e., MGMA-PPIS$_{\text{-EGAT}}$, MGMA-PPIS$_{\text{-EGNN}}$ and MGMA-PPIS$_{\text{-MUSE}}$) of MGMA-PPIS to compare MGMA-PPIS and its three variants on the training dataset in terms of average AUROC and AUPRC using five-fold cross-validation test, and also compare MGMA-PPIS and its three variants on the independent Test_60 dataset in terms of AUROC and AUPRC. MGMA-PPIS$_{\text{-EGAT}}$ represents using the traditional Graph Convolutional Network (GCN) instead of EGAT in MGMA-PPIS framework. MGMA-PPIS$_{\text{-EGNN}}$ represents using the traditional GCN instead of EGNN in MGMA-PPIS framework. MGMA-PPIS$_{\text{-MUSE}}$ represents that removing the multi-scale attention network from MGMA-PPIS framework, but using the same graph neural network architecture. Table 4 shows the results of MGMA-PPIS and its three variants, from which we can see that the AUROC and AUPRC of MGMA-PPIS are higher than that of its variants, demonstrating that all the proposed components for building MGMA-PPIS are valid and contribute to the final performance of MGMA-PPIS. Specifically, EGNN makes the greatest contribution to improving the performance of MGMA-PPIS.

Table 4. Results of MGMA-PPIS and its three variants on the validation and Test_60 datasets

Method	Validation dataset		Test_60 dataset	
	AUROC	AUPRC	AUROC	AUPRC
MGMA-PPIS$_{\text{-MUSE}}$	0.878	0.617	0.884	0.640
MGMA-PPIS$_{\text{-EGAT}}$	0.886	0.649	0.883	0.607
MGMA-PPIS$_{\text{-EGNN}}$	0.820	0.495	0.825	0.504
MGMA-PPIS	**0.887**	**0.648**	**0.901**	**0.673**

3.3. Effectiveness of protein structure features

In our MGMA-PPIS, we extract the protein sequence features and its structure features. The sequence features are derived from PSSM and HMM, while the structural features include DSSP, AF, and PPE. Given that the importance of PSSM, HMM and DSSP have been extensively validated in previous studies [16], we do not re-evaluate these features. Here we investigate the impact of protein structure features (i.e., AF and PPE) on MGMA-PPIS by combining the sequence features (i.e., HMM+PSSM+DSSP) with AF and PPE structure features. The results of MGMA-PPIS with different structure features are shown in Table 5, from which we can see that protein structure features (i.e., AF and PPE) can effectively improve the performance of PPI site prediction. For

example, the AUROC and AUPRC of MGMA-PPIS with HMM+PSSM+DSSP+AF combination features on Test_60 dataset are 0.042, 0.094 higher than that of HMM+PSSM+DSSP combination features, respectively. The AUROC and AUPRC of MGMA-PPIS with HMM+PSSM+DSSP+AF+PPE combination features on Test_60 dataset are 0.011, 0.026 higher than that of HMM+PSSM+DSSP+AF combination features, respectively. Thus, in this work we adopt the combination features of HMM+PSSM+DSSP+AF+PPE in MGMA-PPIS framework to predict PPI sites.

Table 5. Results of MGMA-PPIS with different combination features on the validation and Test_60 datasets

Combination features	Validation dataset		Test_60 dataset	
	AUROC	AUPRC	AUROC	AUPRC
HMM+PSSM+DSSP	0.833	0.520	0.848	0.553
HMM+PSSM+DSSP+AF	0.885	0.641	0.890	0.647
HMM+PSSM+DSSP+AF+PPE	0.887	0.648	0.901	0.673

3.4. Effectiveness of different loss functions

To mitigate the impact of imbalanced positive and negative sample distributions on MGMA_PPIS training, we employ the focal loss instead of the traditional cross-entropy loss, then compare the performance of MGMA-PPIS with these two loss functions on both the validation dataset and the independent test dataset of Test_60. The experimental results are shown in Table 6, from which we can see that the performance of MGMA-PPIS with focal loss function is superior to that with the cross-entropy loss function. For example, the AUROC and AUPRC of MGMA-PPIS with focal loss function on validation dataset are 0.002, 0.008 higher than those with cross-entropy loss function, respectively. These results indicate that focal loss function can mitigate the impact of sample imbalance on model training.

Table 6. Results of MGMA-PPIS with different loss functions on the validation and Test_60 datasets

Loss function	Validation dataset		Test_60 dataset	
	AUROC	AUPRC	AUROC	AUPRC
Cross-entropy loss	0.885	0.640	0.896	0.660
Focal loss	0.887	0.648	0.901	0.673

3.5. Running time analysis

We compare the running time of our MGMA-PPIS with AGAT-PPIS and GHGPR-PPIS methods on the validation dataset. These two comparation methods have the best prediction results among

all comparison methods. As shown in Figure 2, we can see that the running time of our MGMA-PPIS is far lower than that of AGAT-PPIS and GHGPR-PPIS methods, indicating that our MGMA-PPIS has excellent computational efficiency.

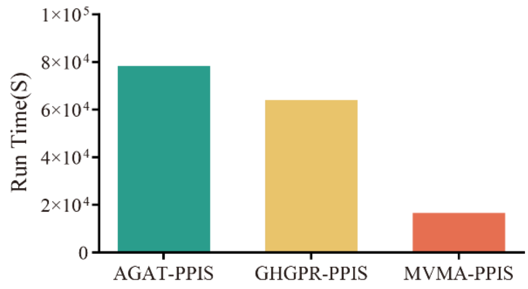

Figure 2 The running time of AGAT-PPIS, GHGPR-PPIS, and MGMA-PPIS on the validation dataset.

3.6. Case study

Through the study of specific protein samples, we aim to further demonstrate the outstanding capability of the MGMA-PPIS in PPI site prediction. Table 7 and Figure 3 present two case study results (i.e., PDB ID: 2yc2, chain A; PDB ID: 3tu3, chain A) of MGMA-PPIS with AGAT-PPIS and GHGPR-PPIS methods from Test_60 dataset. As shown in Table 7 and Figure 3, we can see that our MGMA-PPIS significantly outperforms AGAT-PPIS and GHGPR-PPIS. More protein prediction results are presented in Table S4 and Figure S3. These results show that MGMA-PPIS can effectively reduce the number of false positive sites, thereby improving the performance of PPI site prediction.

Table 7. Results of MGMA-PPIS, GHGPR-PPIS and AGAT-PPIS on a Specific Proteins (PDB ID: 2yc2, Chain A and PDB ID: 3tu3, chain A)

**PDB ID**	**Methods**	**TP**	**TN**	**FP**	**FN**
2yc2, Chain A	AGAT-PPIS	24	82	15	11
	GHGPR-PPIS	22	83	14	13
	MGMA-PPIS	25	95	2	10
3tu3, chain A	AGAT-PPIS	20	48	28	23
	GHGPR-PPIS	23	55	21	20
	MGMA-PPIS	24	59	17	19

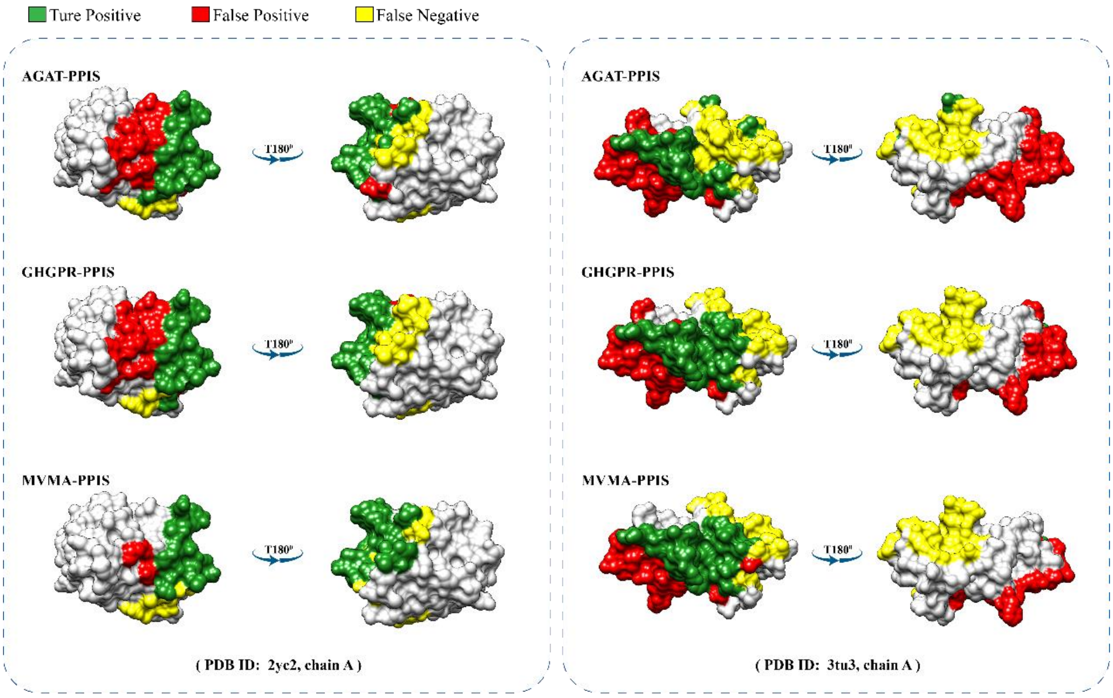

Figure 3 Visualization of prediction results of AGAT-PPIS, GHGPR-PPIS, and MGMA-PPIS on protein 2yc2 Chain A and protein 3tu3 chain A.

4. Conclusion

In this work, we develop an innovative MGMA-PPIS method for high-precision prediction PPI sites. MGMA-PPIS integrates global node features extracted with EGNN and multi-scale local node features extracted with EGAT at different neighborhood scales, thereby constructing a multi-view graph feature representation, then employs a multi-scale attention network (MUSE) to deeply fuse these features across multiple scales, and enabling the precise construction of embedding representations for amino acid nodes within protein graphs. Through a series of comparative experiments, we demonstrate that MGMA-PPIS outperforms other existing methods, and exhibits superior generalization ability across all independent test sets.

Although our MGMA-PPIS has good predictive performance, it has the following limitation. As a structure-based method, MGMA-PPIS requires protein tertiary structure information. while the number of proteins with known tertiary structures is relatively limited, which to some extent restricts the MGMA-PPIS applicability. Looking ahead, future work may explore the integration of protein sequence and structure pre-trained models into the feature extraction process to enrich and diversify the extracted features, thereby further enhancing the performance of PPI site prediction. Moreover, the architecture and methodology proposed in this work are not limited to the prediction of PPI sites, but can also be extended to predict other types of protein binding sites, such as DNA-binding site and drug-binding sites, offering new ideas and approaches for related research fields.

Code and Data Availability

The source code and datasets used in this work can be downloaded from

https://github.com/NWPU-903PR/MGMA-PPIS.

Supplementary Data

Supplementary data are available online.

Funding

This work was supported by National Natural Science Foundation of China (Grant Nos. 62173271, 62473312, 62172338).

Declaration of competing interest

The authors declare that they have no known competing financial interests or personal relationships that could have appeared to influence the work reported in this paper.

References:

[1]. Zhang, J. and L. Kurgan, Review and comparative assessment of sequence-based predictors of protein-binding residues. Briefings in bioinformatics, 2018. 19(5): p. 821-837.

[2]. Berggård, T., S. Linse and P. James, Methods for the detection and analysis of protein–protein interactions. Proteomics, 2007. 7(16): p. 2833-2842.

[3]. Li, X., et al., Network-based methods for predicting essential genes or proteins: a survey. Briefings in bioinformatics, 2020. 21(2): p. 566-583.

[4]. Orii, N. and M.K. Ganapathiraju, Wiki-pi: a web-server of annotated human protein-protein interactions to aid in discovery of protein function. PloS one, 2012. 7(11): p. e49029.

[5]. Soleymani, F., et al., Protein–protein interaction prediction with deep learning: A comprehensive review. Computational and Structural Biotechnology Journal, 2022. 20: p. 5316-5341.

[6]. Lu, H., et al., Recent advances in the development of protein–protein interactions modulators: mechanisms and clinical trials. Signal transduction and targeted therapy, 2020. 5(1): p. 213.

[7]. Qi, R. and Q. Zou, Trends and potential of machine learning and deep learning in drug study at single-cell level. Research, 2023. 6: p. 0050.

[8]. Kong, L., et al., Alcoholic fatty liver disease inhibited the co-expression of Fmo5 and PPARα to activate the NF-κB signaling pathway, thereby reducing liver injury via inducing gut microbiota disturbance. Journal of Experimental & Clinical Cancer Research, 2021. 40: p. 1-18.

[9]. Giot, L., et al., A protein interaction map of Drosophila melanogaster. science, 2003. 302(5651): p. 1727-1736.

[10]. Murakami, Y. and K. Mizuguchi, Applying the Naïve Bayes classifier with kernel density estimation to the prediction of protein–protein interaction sites. Bioinformatics, 2010. 26(15): p. 1841-1848.

[11]. Chen, C., et al., LightGBM-PPI: Predicting protein-protein interactions through LightGBM with multi-information fusion. Chemometrics and Intelligent Laboratory Systems, 2019. 191: p. 54-64.

[12]. Northey, T.C., A. Barešić and A.C. Martin, IntPred: a structure-based predictor of protein–protein interaction sites. Bioinformatics, 2018. 34(2): p. 223-229.

[13]. Jiao, S., et al., iTTCA-RF: a random forest predictor for tumor T cell antigens. Journal of translational medicine, 2021. 19: p. 1-11.

[14]. Chen, C., et al., Improving protein-protein interactions prediction accuracy using XGBoost feature selection and stacked ensemble classifier. Computers in biology and medicine, 2020. 123: p. 103899.

[15]. Deng, A., et al., Developing computational model to predict protein-protein interaction sites based on the XGBoost algorithm. International journal of molecular sciences, 2020. 21(7): p. 2274.

[16]. Zhou, Y., Y. Jiang and Y. Yang, AGAT-PPIS: a novel protein–protein interaction site predictor based on augmented graph attention network with initial residual and identity mapping. Briefings in bioinformatics, 2023. 24(3): p. bbad122.

[17]. Zeng, M., et al., Protein–protein interaction site prediction through combining local and global features with deep neural networks. Bioinformatics, 2020. 36(4): p. 1114-1120.

[18]. Khan, S.H., H. Tayara and K.T. Chong, ProB-site: protein binding site prediction using local features. Cells, 2022. 11(13): p. 2117.

[19]. Li, Y., G.B. Golding and L. Ilie, DELPHI: accurate deep ensemble model for protein interaction sites prediction. Bioinformatics, 2021. 37(7): p. 896-904.

[20]. Zhang, B., et al., Sequence-based prediction of protein-protein interaction sites by simplified long short-term memory network. Neurocomputing, 2019. 357: p. 86-100.

[21]. Yang, Z., et al., AlphaFold2 and its applications in the fields of biology and medicine. Signal Transduction and Targeted Therapy, 2023. 8(1): p. 115.

[22]. Yuan, Q., et al., Structure-aware protein–protein interaction site prediction using deep graph convolutional network. Bioinformatics, 2022. 38(1): p. 125-132.

[23]. Fu, X., et al., AGF-PPIS: a protein–protein interaction site predictor based on an attention mechanism and graph convolutional networks. Methods, 2024. 222: p. 142-151.

[24]. Zeng, X., et al., Ghgpr-ppis: a graph convolutional network for identifying protein-protein interaction site using heat kernel with generalized pagerank techniques and edge self-attention feature processing block. Computers in Biology and Medicine, 2024. 168: p. 107683.

[25]. Vaswani, A., et al., Attention is all you need. Advances in neural information processing systems, 2017. 30.

[26]. Jin, Q., et al., Free-form tumor synthesis in computed tomography images via richer generative adversarial network. Knowledge-Based Systems, 2021. 218: p. 106753.

[27]. Zhao, G., et al., Muse: Parallel multi-scale attention for sequence to sequence learning. arXiv preprint arXiv:1911.09483, 2019.

[28]. Das, S., U. Pradhan and S.N. Rai, Five years of gene networks modeling in single-cell RNA-sequencing studies: current approaches and outstanding challenges. Current Bioinformatics, 2022. 17(10): p. 888-908.

[29]. Altschul, S.F., et al., Gapped BLAST and PSI-BLAST: a new generation of protein database search programs. Nucleic acids research, 1997. 25(17): p. 3389-3402.

[30]. Remmert, M., et al., HHblits: lightning-fast iterative protein sequence searching by HMM-HMM alignment. Nature methods, 2012. 9(2): p. 173-175.

[31]. Kabsch, W. and C. Sander, Dictionary of protein secondary structure: pattern recognition of hydrogen-bonded and geometrical features. Biopolymers: Original Research on Biomolecules, 1983. 22(12): p. 2577-2637.

[32]. Satorras, V.G., E. Hoogeboom and M. Welling. E (n) equivariant graph neural networks. in International conference on machine learning. 2021: PMLR.

[33]. Kamiński, K., et al., Rossmann-toolbox: a deep learning-based protocol for the prediction and design of cofactor specificity in Rossmann fold proteins. Briefings in bioinformatics, 2022. 23(1): p. bbab371.
[34]. Veličković, P., et al., Graph attention networks. arXiv preprint arXiv:1710.10903, 2017.
[35]. Scarselli, F., et al., The graph neural network model. IEEE transactions on neural networks, 2008. 20(1): p. 61-80.
[36]. Zhao, G., et al., Muse: Parallel multi-scale attention for sequence to sequence learning. arXiv preprint arXiv:1911.09483, 2019.
[37]. Howard, A.G., et al., Mobilenets: Efficient convolutional neural networks for mobile vision applications. arXiv preprint arXiv:1704.04861, 2017.
[38]. Wu, F., et al., Pay less attention with lightweight and dynamic convolutions. arXiv preprint arXiv:1901.10430, 2019.

Click here to access/download
Supplementary Material
Supplementary Material.docx
