# Supplementary material for: MGMA-PPIS: Predicting the protein–protein interaction site with multiview graph embedding and multiscale attention fusion
Source: Gigascience. 2025 Oct 1;14:giaf114. doi: 10.1093/gigascience/giaf114 (PMC12486388; doi:10.1093/gigascience/giaf114)
Supplement: giaf114_Authors_Response_To_Reviewer_Comments_Original_Submission [file giaf114_authors_response_to_reviewer_comments_original_submission.pdf]

Author's Response To Reviewer Comments

Dear Editor and Reviewers,
Thanks for your valuable comments and professional suggestions, which benefit us for improving our manuscript. We have carefully considered all the comments and suggestions, and revised our manuscript accordingly in this version. The revised parts are highlighted (in blue) in this revised manuscript. The detailed point-by-point responses are listed following each of the comments of reviewers and editor. The sentences highlighted in black are the comments of reviewers and editor, and the sentences highlighted in blue are our responses. The full set of responses has been compiled into a Word document entitled Respond to GIGA for MGMA-PPIS-0816, which has been uploaded together with the revised manuscript.

Cookie Preference Center

We use cookies which are necessary to make our site work. We may also use additional cookies to analyze, improve and personalize our content and your digital experience. For more information, see our Cookie Policy.

You may choose not to allow some types of cookies. However, blocking some types may impact your experience of our site and the services we are able to offer. See the different category headings below to find out more or change your settings.

Allow all

Manage Consent Preferences

Strictly Necessary Cookies

Always active

These cookies are necessary for the website to function and cannot be switched off in our systems. They are usually only set in response to actions made by you which amount to a request for services, such as setting your privacy preferences, logging in or filling in forms. You can set your browser to block or alert you about these cookies, but some parts of the site will not then work.

Functional Cookies

These cookies enable the website to provide enhanced functionality and personalisation. They may be set by us or by third party providers whose services we have added to our pages. If you do not allow these cookies then some or all of these services may not function properly.

Performance Cookies

These cookies allow us to count visits and traffic sources so we can measure and improve the performance of our site. They help us to know which pages are the most and least popular and see how visitors move around the site.

Targeting Cookies

These cookies may be set through our site by our advertising partners. They may be used by those companies to build a profile of your interests and show you relevant adverts on other sites. If you do not allow these cookies, you will experience less targeted advertising.

Cookie List

Clear

Apply Cancel

Consent Leg.Interest

Confirm my choices

Powered by onetrust
