# Supplementary material for: MGMA-PPIS: Predicting the protein–protein interaction site with multiview graph embedding and multiscale attention fusion
Source: Gigascience. 2025 Oct 1;14:giaf114. doi: 10.1093/gigascience/giaf114 (PMC12486388; doi:10.1093/gigascience/giaf114)
Supplement: giaf114_Reviewer_1_Report_Original_Submission — Juntao Liu -- 7/26/2025 [file giaf114_reviewer_1_report_original_submission.pdf]

Reviewer Report

Title: MGMA-PPIS: Predicting protein-protein interaction site with multi-view graph embedding and multi-scale attention fusion

Version: Original Submission **Date:** 7/26/2025

Reviewer name: Juntao Liu

Reviewer Comments to Author:

The authors integrate global features (extracted by an equivariant graph neural network with local features (captured by an edge-based graph attention network at multiple neighborhood scales) to represent proteins in a multi-view graph way. A multi-scale attention mechanism is then employed to fuse these features, enabling high-precision prediction of protein-protein interaction sites. The proposed method is novel and well-validated, and the experimental validations are convincing. Overall, this paper is solid work and can be accepted for publication, subject to the following minor corrections
There are inconsistencies in the module labels in Figure 1. For instance, it is unclear whether "MUSE" and "Multi-Scale attEntion" refer to the same module. It is recommended to standardize the naming conventions and terminology to improve the clarity and consistency of the illustration.
The authors employ EGNN to extract global features, adopt EGAT to capture local features at different scales. Although experiments with different combinations of layer numbers have been conducted, the authors should further improve the experimental strategy by systematically investigating how the number of EGAT layers affects model performance under a fixed EGNN depth, in order to more comprehensively validate the effectiveness of the proposed multi-scale modeling approach.
The authors employed the MUSE module and claimed that it outperforms the traditional self-attention mechanism for feature fusion. However, a direct performance comparison between the two approaches is lacking. The authors should provide the comparative results to demonstrate the advantages of MUSE module.
The authors should enhance the ablation study by analyzing the impact of each module on model performance, to more comprehensively validate the effectiveness and necessity of the proposed components.
The authors should carefully proofread the manuscript to correct typographical and formatting issues, such as the improper citation format in "across numerous tasks[xx]". In addition, further language polishing is recommended to improve the overall quality of the manuscript.

Methods

Are the methods appropriate to the aims of the study, are they well described, and are necessary controls included? Choose an item.

Conclusions

Are the conclusions adequately supported by the data shown? Choose an item.

Reporting Standards

Does the manuscript adhere to the journal's guidelines on minimum standards of reporting? Choose an item.

Choose an item.

Statistics

Are you able to assess all statistics in the manuscript, including the appropriateness of statistical tests used? Choose an item.

Quality of Written English

Please indicate the quality of language in the manuscript: Choose an item.

Declaration of Competing Interests

Please complete a declaration of competing interests, considering the following questions:

- Have you in the past five years received reimbursements, fees, funding, or salary from an organisation that may in any way gain or lose financially from the publication of this manuscript, either now or in the future?
- Do you hold any stocks or shares in an organisation that may in any way gain or lose financially from the publication of this manuscript, either now or in the future?
- Do you hold or are you currently applying for any patents relating to the content of the manuscript?
- Have you received reimbursements, fees, funding, or salary from an organization that holds or has applied for patents relating to the content of the manuscript?
- Do you have any other financial competing interests?
- Do you have any non-financial competing interests in relation to this paper?

If you can answer no to all of the above, write 'I declare that I have no competing interests' below. If your reply is yes to any, please give details below.

'I declare that I have no competing interests

I agree to the open peer review policy of the journal. I understand that my name will be included on my report to the authors and, if the manuscript is accepted for publication, my named report including any attachments I upload will be posted on the website along with the authors' responses. I agree for my report to be made available under an Open Access Creative Commons CC-BY license (http://creativecommons.org/licenses/by/4.0/). I understand that any comments which I do not wish to be included in my named report can be included as confidential comments to the editors, which will not be published.

Choose an item.

To further support our reviewers, we have joined with Publons, where you can gain additional credit to further highlight your hard work (see: https://publons.com/journal/530/gigascience). On publication of this paper, your review will be automatically added to Publons, you can then choose whether or not to claim your Publons credit. I understand this statement.

Yes Choose an item.
