# Supplementary material for: MGMA-PPIS: Predicting the protein–protein interaction site with multiview graph embedding and multiscale attention fusion
Source: Gigascience. 2025 Oct 1;14:giaf114. doi: 10.1093/gigascience/giaf114 (PMC12486388; doi:10.1093/gigascience/giaf114)
Supplement: giaf114_Reviewer_2_Report_Original_Submission — Jianmin Wang -- 7/28/2025 [file giaf114_reviewer_2_report_original_submission.pdf]

Reviewer Report

Title: MGMA-PPIS: Predicting protein-protein interaction site with multi-view graph embedding and multi-scale attention fusion

Version: Original Submission **Date:** 7/28/2025

Reviewer name: Jianmin Wang

Reviewer Comments to Author:

The manuscript presents MGMA-PPIS, a graph neural network-based framework for predicting protein-protein interaction sites by integrating multi-view graph embeddings and a multi-scale attention fusion mechanism. While the proposed method demonstrates competitive performance across several benchmark datasets and incorporates multiple architectural innovations, there are several important concerns regarding novelty, design justification, and clarity that need to be thoroughly addressed before the manuscript can be considered for publication.

1. The manuscript lacks a clearly articulated novelty statement. While MGMA-PPIS integrates EGAT, EGNN, and multi-scale attention, these components individually have been used in prior works. The authors should clearly highlight what is fundamentally new about their fusion strategy beyond the aggregation of existing modules. A diagrammatic comparison with prior methods would strengthen this point.
2. The dataset used (AGAT-PPIS) is highly imbalanced, yet the data splitting is not sufficiently detailed. Authors should clarify how the imbalance was handled during training, validation, and testing, and whether any resampling or class weighting strategies were used beyond focal loss.
3. The method shows improved performance metrics, but the biological interpretability of the predictions is not explored. Could the authors include an interpretability analysis (e.g., attention weight visualization, feature importance, or case-specific insights) to validate if the model is learning biologically meaningful features?
4. Inconsistent usage of the model name (e.g., "MGMA-PPIS-PPIS") appears multiple times, which may confuse readers. Additionally, terms like "animo acid" (should be "amino acid") and other typos are present. Please thoroughly revise the manuscript for grammatical and terminological consistency.

Methods

Are the methods appropriate to the aims of the study, are they well described, and are necessary controls included? Choose an item.

Conclusions

Are the conclusions adequately supported by the data shown? Choose an item.

Reporting Standards

Does the manuscript adhere to the journal's guidelines on minimum standards of reporting? Choose an item.

Choose an item.

Statistics

Are you able to assess all statistics in the manuscript, including the appropriateness of statistical tests used? Choose an item.

Quality of Written English

Please indicate the quality of language in the manuscript: Choose an item.

Declaration of Competing Interests

Please complete a declaration of competing interests, considering the following questions:

- Have you in the past five years received reimbursements, fees, funding, or salary from an organisation that may in any way gain or lose financially from the publication of this manuscript, either now or in the future?
- Do you hold any stocks or shares in an organisation that may in any way gain or lose financially from the publication of this manuscript, either now or in the future?
- Do you hold or are you currently applying for any patents relating to the content of the manuscript?
- Have you received reimbursements, fees, funding, or salary from an organization that holds or has applied for patents relating to the content of the manuscript?
- Do you have any other financial competing interests?
- Do you have any non-financial competing interests in relation to this paper?

If you can answer no to all of the above, write 'I declare that I have no competing interests' below. If your reply is yes to any, please give details below.

no

I agree to the open peer review policy of the journal. I understand that my name will be included on my report to the authors and, if the manuscript is accepted for publication, my named report including any attachments I upload will be posted on the website along with the authors' responses. I agree for my report to be made available under an Open Access Creative Commons CC-BY license (http://creativecommons.org/licenses/by/4.0/). I understand that any comments which I do not wish to be included in my named report can be included as confidential comments to the editors, which will not be published.

Choose an item.

To further support our reviewers, we have joined with Publons, where you can gain additional credit to further highlight your hard work (see: https://publons.com/journal/530/gigascience). On publication of this paper, your review will be automatically added to Publons, you can then choose whether or not to claim your Publons credit. I understand this statement.

Yes Choose an item.
